# Unraveling the molecular links between benzopyrene exposure, NASH, and HCC: an integrated bioinformatics and experimental study

**DOI:** 10.1038/s41598-023-46440-1

**Published:** 2023-11-22

**Authors:** Zheming Yang, Jiayin Li, Haixu Song, Zhu Mei, Xiaodong Jia, Xiaoxiang Tian, Chenghui Yan, Yaling Han

**Affiliations:** 1 College of Medicine and Biological Information Engineering, Northeastern University, Shenyang, 110167 Liaoning China; 2 State Key Laboratory of Frigid Zone Cardiovascular Diseases (SKLFZCD), Cardiovascular Research Institute and Department of Cardiology, General Hospital of Northern Theater Command, Shenyang, 110016 China

**Keywords:** Biotechnology, Cancer, Computational biology and bioinformatics

## Abstract

Benzopyrene (B[a]P) is a well-known carcinogen that can induce chronic inflammation and fibrosis in the liver, leading to liver disease upon chronic exposure. Nonalcoholic steatohepatitis (NASH) is a chronic liver condition characterized by fat accumulation, inflammation, and fibrosis, often resulting in hepatocellular carcinoma (HCC). In this study, we aimed to investigate the intricate connections between B[a]P exposure, NASH, and HCC. Through comprehensive bioinformatics analysis of publicly available gene expression profiles, we identified differentially expressed genes (DEGs) associated with B[a]P exposure, NASH, and liver cancer. Furthermore, network analysis revealed hub genes and protein–protein interactions, highlighting cellular metabolic dysfunction and disruption of DNA damage repair in the B[a]P-NASH-HCC process. Notably, HSPA1A and PPARGC1A emerged as significant genes in this pathway. To validate their involvement, we conducted qPCR analysis on cell lines and NASH mouse liver tissues and performed immunohistochemistry labeling in mouse and human HCC liver sections. These findings provide crucial insights into the potential regulatory mechanisms underlying benzopyrene-induced hepatotoxicity, shedding light on the pathogenesis of B[a]P-associated NASH and HCC. Moreover, our study suggests that HSPA1A and PPARGC1A could serve as promising therapeutic targets. Enhancing our understanding of their regulatory roles may facilitate the development of targeted therapies, leading to improved patient outcomes.

## Introduction

Liver diseases have been linked to exposure to a variety of stressors that can impact the body systemically. At present, with the rapid development of economy and industrialization, many chemicals are released into the environment^1^. Of particular concern are certain chemicals known for their hepatotoxic effects, including endocrine disrupters, pesticides, and substances such as perfluoroalkyl, polyfluoroalkyl, and polycyclic aromatic hydrocarbons (PAHs)^2,3^. However, it is important to note that currently, there are limited effective detect and treatment options available for liver diseases caused by these pollutants^4,5^. Therefore, understanding of these hepatotoxic agents are crucial for public health.

Among these substances, benzopyrene stands out as an extremely hazardous and widely prevalent environmental contaminant. Benzopyrene (B[a]P), a PAH compound, is commonly produced during the incomplete combustion of organic materials and is found practically everywhere in the environment, including air, water, and soil^6,7^. The global concern surrounding benzopyrene arises from its strong association with the development of hepatocellular carcinoma (HCC), which remains a major cause of cancer-related deaths worldwide^8,9^. Despite its strong association with HCC, However, the effects of B[a]P exposure on progression of HCC and the potential mechanisms remains largely uninvestigated^10^. In addition to its role in HCC pathogenesis, growing evidence suggests that B[a]P exposure is also implicated in the onset of non-alcoholic steatohepatitis (NASH)^11^. Therefore, it is crucial to explore treatment strategies, and conduct research on the mechanisms of toxicity associated with B[a]P.

NASH is a chronic liver condition caused by hepatic steatosis, or fat accumulation in the liver^12^. Due to the fact that hepatic fibrosis and cirrhosis are frequent side effects of this condition, NASH has been identified as a substantial risk factor for the development of HCC^13,14^. NASH is primarily caused by oxidative stress, insulin resistance, and inflammation, all of which can induce hepatic steatosis and fibrosis^15,16^. Furthermore, the accumulation of genetic and epigenetic changes in the liver can accelerate the progression of NASH to HCC^17^.

The development of B[a]P -induced liver disorders including NASH and HCC has been studied extensively, and integrated bioinformatics tools have been found to be beneficial in finding putative hub genes and pathways implicated in this process. These methods make use of high-throughput technology to examine huge molecular profile datasets, allowing for a thorough evaluation of the pathways and gene networks implicated in disease progression^18^.

Understanding the molecular mechanisms underlying B[a]P-induced NASH and HCC is critical for discovering novel treatment targets and biomarkers for prognostic evaluation. This strategy has the potential to direct the development of novel preventative treatments and targeted therapeutics for NASH and HCC by identifying important genes and molecular pathways implicated in B[a]P toxicity. Overall, this study establishes a paradigm for understanding the molecular pathways behind B[a]P, NASH, and HCC, and gives vital insight into the link between these disorders.

Three microarray datasets from the GEO database (GSE127791, GSE164760, and GSE146049) were used to examine DEGs in this investigation. Survival analysis was performed using data from the TCGA-LIHC cohort. GSE127791 is an RNA-seq by array dataset of B[a]P-treated HepaRG cells that can be used to examine B[a]P-induced hepatotoxicity. GSE164760 is an RNA-seq dataset of NASH patients as well as healthy individuals, whereas GSE146049 is an RNA-seq dataset of HCC patients' tumor and non-tumor tissue. Our goal using bioinformatics analysis was to provide new insights into the molecular pathways underpinning B[a]P-induced NASH and HCC formation and progression, which will lead to novel targets for HCC diagnosis and treatment.

## Results

### Identification and analysis of DEGs

**Figure 1:**
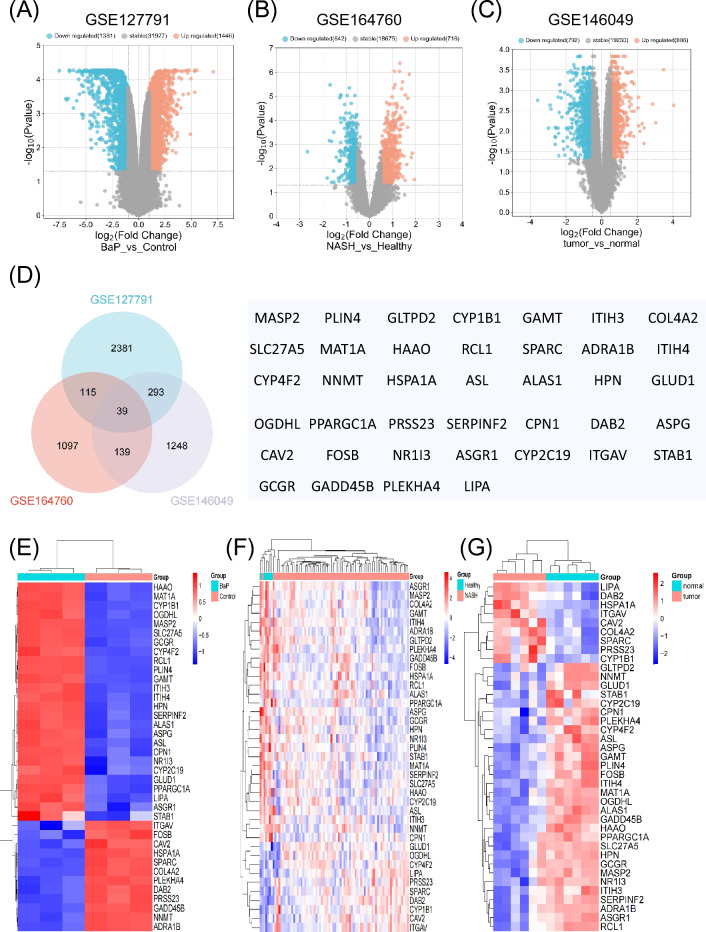

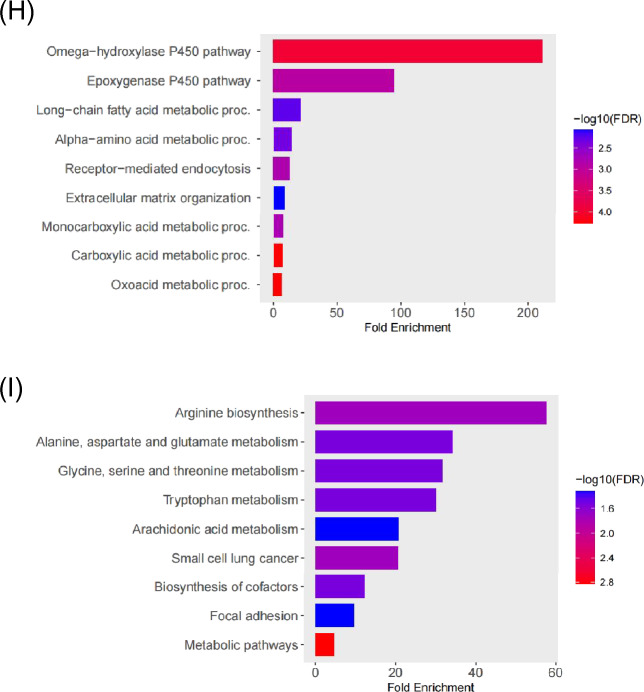
Differentially expressed genes (DEGs) and functional enrichment analysis in B[a]P exposed, NASH and liver cancer datasets. (**A**–**C**) Volcano plot of DEGs in GSE127791, GSE164760 and GSE146049 datasets. Red color indicated up-regulated genes and blue color indicated downregulated genes. (**D**) Venn diagram of overlapping DEGs among three GEO datasets. (**E–G**) Heat maps of overlapping DEGs in GSE127791, GSE164760 and GSE146049 datasets. (**H**) Gene ontology (GO) biological processes (BP) enrichment analysis of 39 overlapping DEGs. (**I**) Kyoto encyclopedia of genes and genomes (KEGG) pathway analysis of 39 overlapping DEGs.

The GEO database's microarray data were used in this investigation. |log2(FC)|> 0.5 and *p* < 0.05 were the selection criterion for differentially expressed genes (DEGs). The SRPLOT tools were used to create volcano plots to display the variations in gene expression. GSE127791, GSE164760, and GSE146049 were the three accession numbers that were picked for analysis. There were 1446, 716, and 886 up-regulated genes in these datasets, and 1381, 642, and 792 down-regulated genes, respectively, according to the volcano plots (Fig. 1A–C). A Venn diagram was created to find common DEGs among the selected datasets. It was discovered that GSE127791, GSE164760, and GSE146049 shared 39 DEGs (Fig. 1D). A heat map was also constructed to depict the expression patterns of these DEGs in the selected datasets (Fig. 1E–G).

The combined DEGs' biological categorization was looked at. According to GO and KEGG pathway studies, these DEGs showed high enrichment in a number of functional categories, such as amino acid synthesis and metabolism, fatty acid metabolism, cellular endocytosis, and extracellular matrix (Fig. 1H). Additionally, the KEGG pathway analysis showed that the DEGs were primarily linked to lipid metabolism and the manufacture of different amino acids (Fig. 1I). Disruptions in cellular metabolic pathways were suggested by the network of DEGs linked with HCC, NASH, and B[a]P.

### Identification and analysis of hub genes

**Figure 2:**
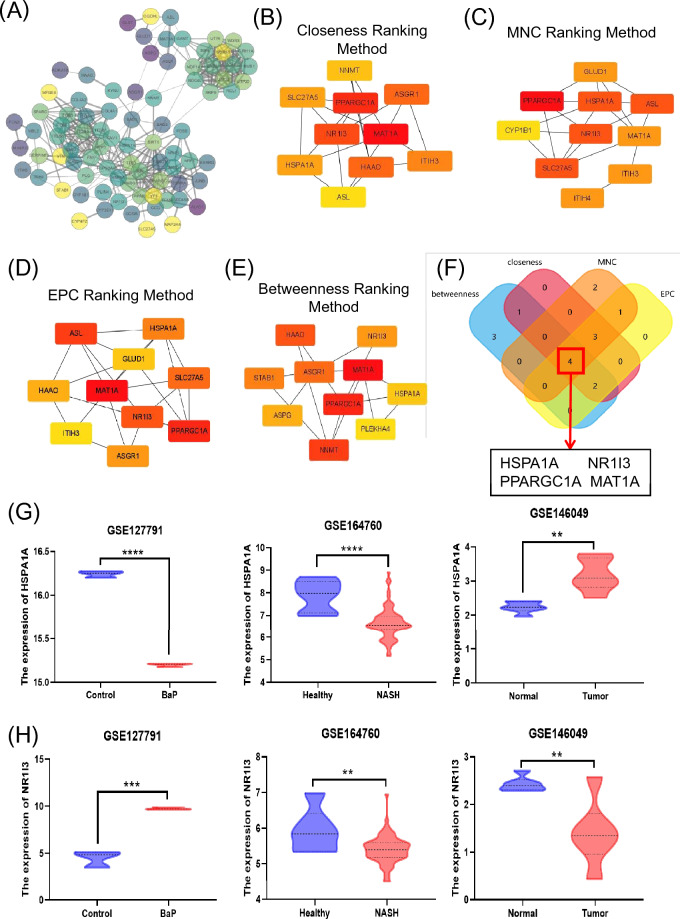

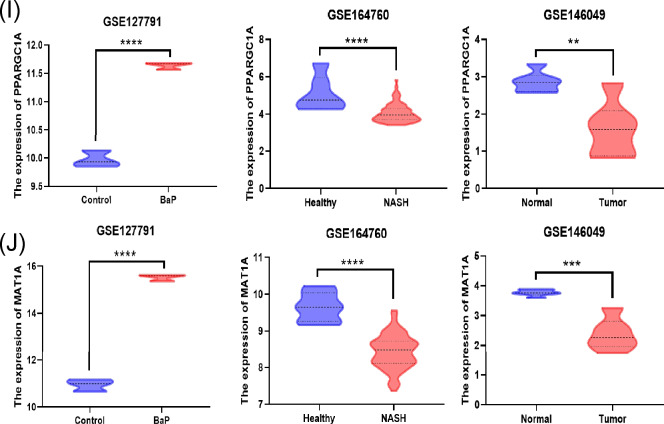
Identification of Hub genes in overlapping DEGs among three GEO datasets. (**A**) Protein–protein interaction of the overlapping DEGs. (**B**–**E**) The closeness ranking method, the maximal neighborhood component (MNC) ranking method, the edge percolated component (EPC) ranking method, and the betweenness ranking method for hub genes identification. (**F**) Venn diagram for identifying hub genes among different ranking methods. (**G**) The expression of HSPA1A in three GEO datasets. (**H**) The expression of NR1I3 in three GEO datasets. (**I**) The expression of PPARGC1A in three GEO datasets. (**J**) The expression of MAT1A in three GEO datasets. Red color represents highest degree, and orange color represents intermedia degree, and yellow color represents lowest degree. ** *p* < 0.01; *** *p* < 0.001; **** *p* < 0.0001.

The STRING online tool was used to build the PPI network of the overlapped DEGs (Fig. 2A). Using Cytoscape v3.10.0, the resulting query protein was visualised as a PPI network, and the hub genes were identified using the cytoHubba tool, which returned a list of the top-ranked proteins. In CytoHubba, four ranking approaches were used to discover the hub genes: proximity ranking, maximal neighbourhood component (MNC) ranking, edge percolated component (EPC) ranking, and betweenness ranking. The top ten hub genes chosen by each approach (Fig. 2B–E). Heat Shock Protein Family A (Hsp70) Member 1A (HSPA1A), Nuclear Receptor Subfamily 1 Group I Member 3 (NR1I3), PPARG Coactivator 1 Alpha (PPARGC1A), and Methionine Adenosyltransferase 1A (MAT1A) were discovered as four common interacting hub genes by the intersection of these four approaches (Fig. 2F). HSPA1A gene mRNA expression was shown to be lower in B[a]P and NASH-related datasets, but higher in HCC (Fig. 2G). The mRNA expression of the other three genes, on the other hand, increased in B[a]P-related datasets while decreasing in NASH and HCC (Fig. 2H–J). Finally, the core genes linked to B[a]P-NASH-HCC were HSPA1A, NR1I3, PPARGC1A, and MAT1A. This implies that cells attempt to lower the expression of detrimental genes while increasing the expression of helpful genes in order to fight the damage caused by disease progression, but this is ultimately irreversible.

### Functional enrichment analysis of proteins interacting with four genes

**Figure 3:**
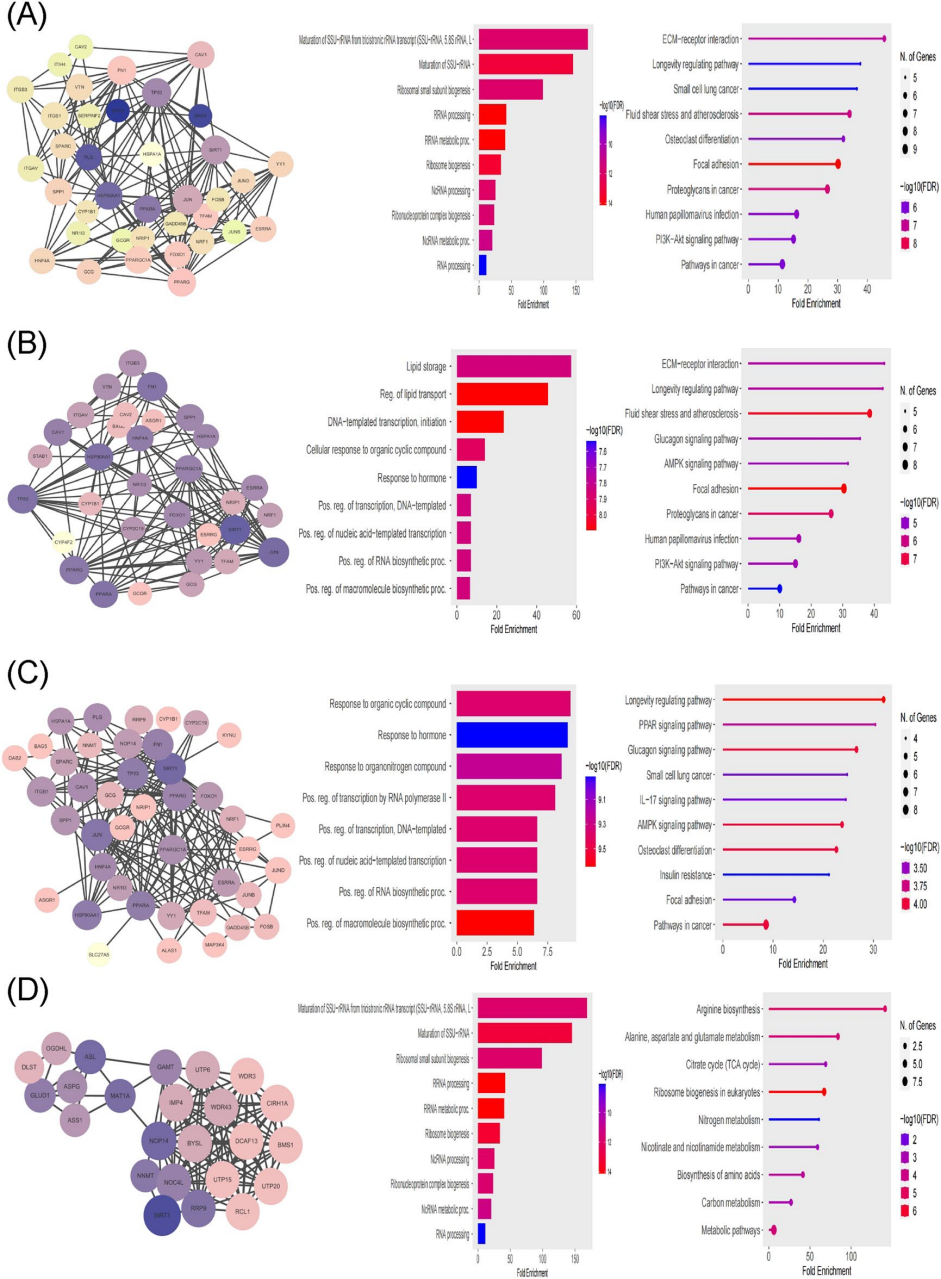
Functional enrichment analysis of proteins interacting with four genes. (**A**) Protein interaction network of HSPA1A. BP and KEGG enrichment analysis of protein interaction network of HSPA1A. (**B**) Protein interaction network of NR1I3. BP and KEGG enrichment analysis of protein interaction network of NR1I3. (**C**) Protein interaction network of PPARGC1A. BP and KEGG enrichment analysis of protein interaction network of PPARGC1A. (**D**) Protein interaction network of MAT1A. BP and KEGG enrichment analysis of protein interaction network of MAT1A.

According to the GO-BP database, the functional enrichment analysis of proteins interacting with the HSPA1A gene revealed a predominance of pathways involved in the regulation of ribosomal RNA generation, maturation, and transport. Furthermore, the KEGG analysis revealed that the majority of these proteins were enriched in extracellular matrix receptor and cancer-related pathways (Fig. 3A). In GO-BP, the proteins that interact with NR1I3 were discovered to be involved in lipid deposition and DNA transcription processes. In terms of KEGG enrichment, the most enriched pathway for proteins interacting with NR1I3 was extracellular matrix receptor interaction (Fig. 3B). Following that, an enrichment analysis of proteins interacting with the PPARGC1A gene was carried out. The bulk of these proteins were shown to be enriched in hormone response, organic cyclic compounds, and organic nitrogen compounds pathways. According to the KEGG analysis, the majority of these proteins were shown to be enriched in the PPAR signaling pathway and the glucagon signaling pathway (Fig. 3C). Furthermore, a GO-BP enrichment analysis of proteins interacting with the MAT1A gene revealed that a high fraction of these proteins were enriched in biological processes such as short ribosomal RNA and non-coding RNA. According to the KEGG study, a significant number of these proteins enriched in the biosynthesis and metabolism of various amino acids (Fig. 3D). We used TCGA-LIHC patient samples for additional validation to guarantee the veracity of the enrichment results. The GO-BP analysis indicated significantly significant enrichment in pathways such as lipid modification, protein maturation, protein localisation at the cell periphery, and intracellular receptor signaling in HSPA1A high expression patients (Table S1). Protein quality control for misfolded or incompletely synthesized proteins, proton motive force driven ATP synthesis, establishment of protein localization to mitochondrial membrane, nuclear membrane reassembly, maturation of rRNA, viral translation, lysosomal lumen acidification, regulation of mitochondrial translation and 2-Oxoglutarate metabolic process were all found to be enriched in NR1I3 low expression patients (Table S2). PPARGC1A low expression patients displayed highly significant enrichment in pathways involving protein quality control for misfolded or incompletely synthesized proteins, proton motive force driven ATP synthesis, ribosome assembly, maturation of rRNA, rRNA transcription, rvesicle tethering, response to misfolded protein, establishment of protein localization to mitochondrial membrane and branched chain amino acid catabolic process (Table S3). Finally, patients with low MAT1A expression showed highly significant enrichment in pathways such as viral translation, cristae formation, negative regulation of Torc1 signaling, rRNA transcript, cleavage involved in rRNA processing, RNA surveillance, regulation of protein neddylation, mitochondrial RNA processing, ribosomal subunit export from nucleus and positive regulation of mitochondrial translation (Table S4). The KEGG analysis of patients with high HSPA1A expression revealed enrichment in glycosphingolipid metabolism, the ErbB signaling pathway, long-term potentiation (LTP), the adipocytokine signaling pathway, leukocyte transendothelial migration, regulation of the actin cytoskeleton, focal adhesion, and butanoate metabolism (Table S5). Similarly, protein export, RNA degradation, aminoacyl tRNA biosynthesis, DNA replication, glycosylphosphatidylinositol (GPI) anchor biosynthesis, base excision repair, one carbon pool by folate, mismatch repair, glyoxylate and dicarboxylate metabolism and glycine serine and threonine metabolism were all enriched in the NR1I3 low expression group (Table S6). The KEGG analysis revealed enrichment in pathways such as RNA degradation, lysine degradation, protein export, citrate cycle TCA cycle, aminoacyl tRNA biosynthesis, fatty acid metabolism, DNA replication, nucleotide excision repair, base excision repair and propanoate metabolism for PPARGC1A low expression patients (Table S7). Finally, the MAT1A low expression group enriched in protein export, aminoacyl tRNA biosynthesis, one carbon pool by folate and mismatch repair (Table S8). Furthermore, we found significant differences in clinicopathological characteristics (age, gender, fibrosis Ishak score, pathologic T, and race) between HSPA1A, NR1I3, PPARGC1A, and MAT1A gene expressions and hepatocellular carcinoma patients (Tables S9–S12).

### Four hub genes are related to immune cell infiltration

**Figure 4:**
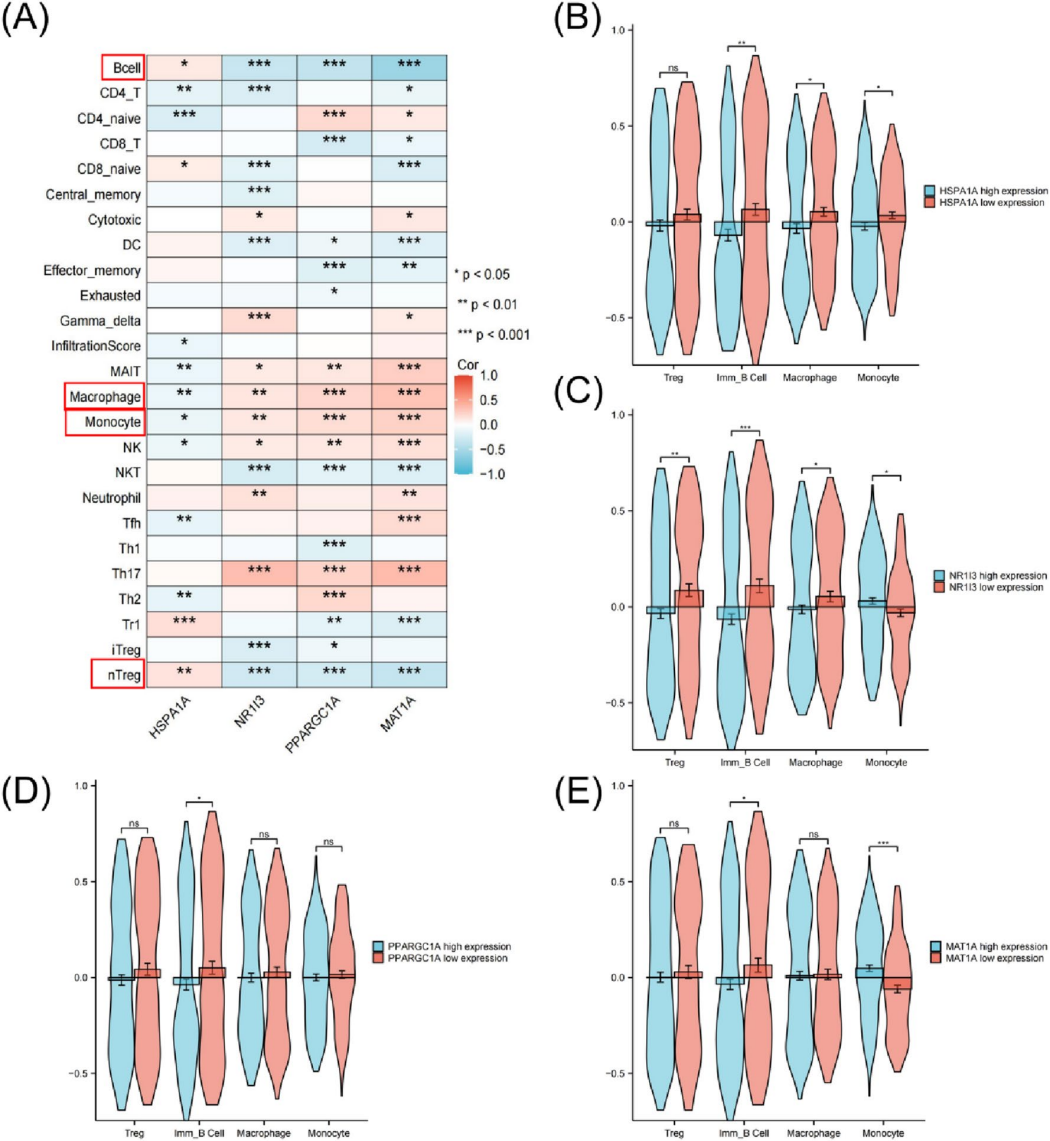
Four hub genes are related immune cell infiltration. (**A**) The correlation betweenimmune cell infiltration and the expression of HSPA1A, NR1I3, PPARGC1A and MAT1A in TCGA-LIHC cohort. (**B**) Comparison of ssGSEA scores between HSPA1A high expression group and HSPA1A low expression group in TCGA-LIHC cohort. (**C**) Comparison of ssGSEA scores between NR1I3 high expression group and NR1I3 low expression group in TCGA-LIHC cohort. (**D**) Comparison of ssGSEA scores between PPARGC1A high expression group and PPARGC1A low expression group in TCGA-LIHC cohort. (**E**) Comparison of ssGSEA scores between MAT1A high expression group and MAT1A low expression group in TCGA-LIHC cohort. * *p* < 0.05; ** *p* < 0.01; *** *p* < 0.001.

There is a link between four hub genes and immune infiltration. To investigate the roles of HSPA1A, NR1I3, PPARGC1A, and MAT1A genes in the immune microenvironment of HCC patients, we evaluated inflammatory cell infiltration. HSPA1A, NR1I3, PPARGC1A and MAT1A genes were found to be highly positively correlated with nTreg cells, B cells, macrophages and monocytes in TCGA LIHC (Fig. 4A). The ssGSEA scores of immune cells were compared between patients with HSPA1A, NR1I3, PPARGC1A and MAT1A high and low expression in TCGA-LIHC patients, and only immune B cells were found to be significantly correlated between the four groups (Fig. 4B–D).

### Survival analysis of HSPA1A, NR1I3, PPARGC1A and MAT1A in TCGA-LIHC cohort

**Figure 5:**
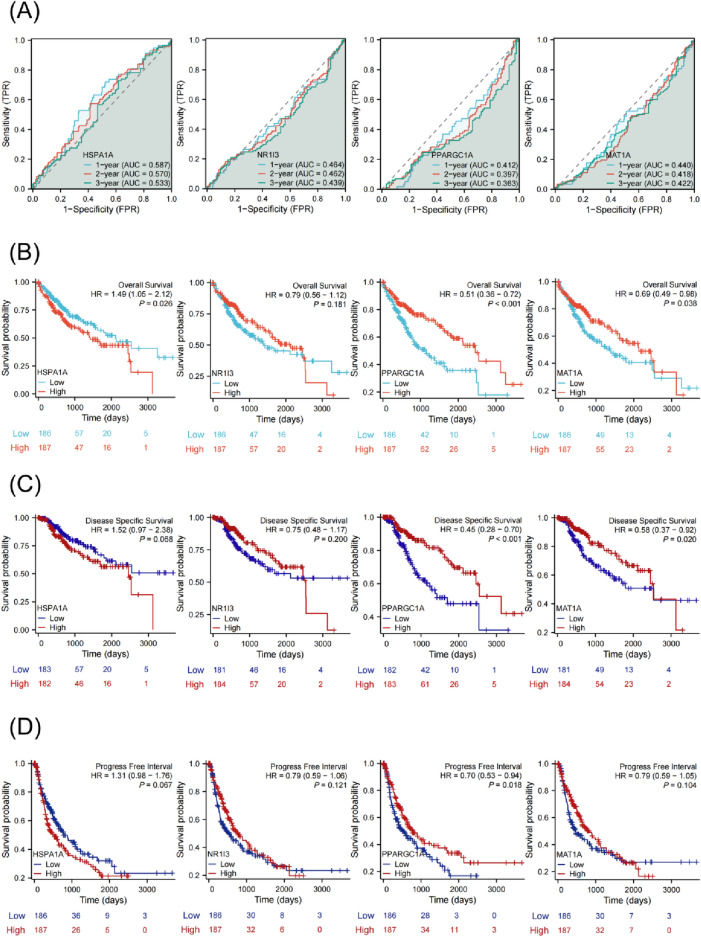
Survival analysis of four hub genes in TCGA-LIHC cohort. (**A**) ROC curves of TCGA-LIHC cohort. The AUC values shown the predictive efficiency of HSPA1A, NR1I3, PPARGC1A and MAT1A on the 1-, 2-, and 3-years survival rate. (**B**) The overall survival analysis of HSPA1A, NR1I3, PPARGC1A and MAT1A in TCGA-LIHC cohort. (**C**) The overall survival analysis of HSPA1A, NR1I3, PPARGC1A and MAT1A in TCGA-LIHC cohort. (**D**) The disease-specific survival analysis of HSPA1A, NR1I3, PPARGC1A and MAT1A in TCGA-LIHC cohort.

**Figure 6:**
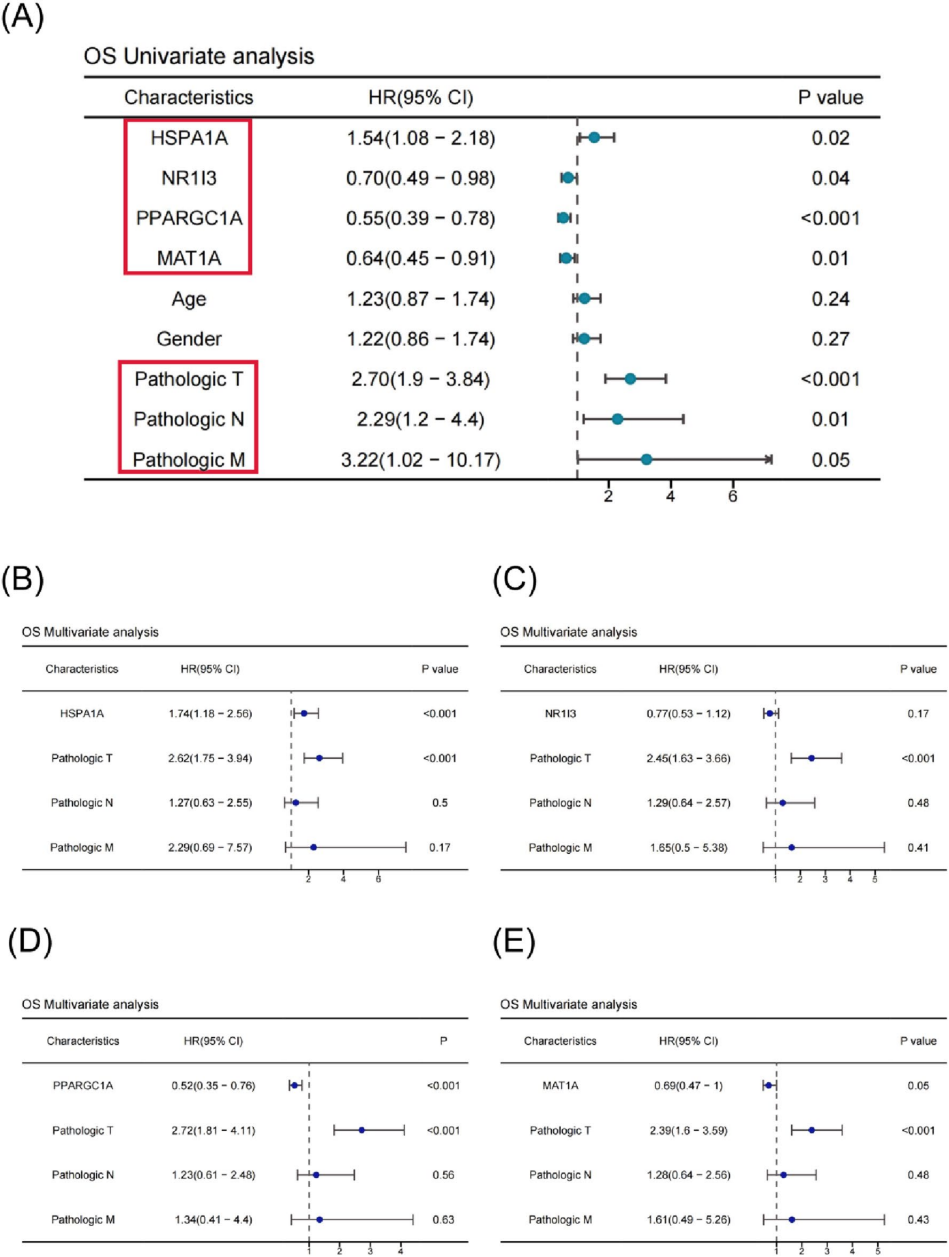
Cox analyses of four hub genes in TCGA-LIHC cohort. (**A**) Univariate Cox analyses evaluated the independent prognostic value of HSPA1A, NR1I3, PPARGC1A and MAT1A in terms of OS in TCGA-LIH. (**B**–**E**) Multivariate Cox analyses evaluated the independent prognostic value of HSPA1A, NR1I3, PPARGC1A and MAT1A in terms of OS in TCGA-LIHC.

To test the prognostic usefulness of these markers for patient survival, a time-dependent receiver-operating characteristic (ROC) analysis was undertaken. The area under the ROC curve (AUC) for 1, 2, and 3-year overall survival for the HSPA1A, NR1I3, PPARGC1A, and MAT1A genes was calculated (Fig. 5A). ROC and Kaplan–Meier curves were used to assess prognostic capacity. The overall survival curves of the other three genes, with the exception of the NR1I3, were significantly different between the high-risk and low-risk groups (Fig. 5B–D). The disease-specific survival and disease-free interval curves for the PPARGC1A gene also differed considerably between the high-risk and low-risk groups. On TCGA-LIHC patients, univariate and multivariate Cox regression analysis were performed to see if the risk score predicted prognosis independently. According to the findings of the univariate Cox regression analysis, a higher risk score was highly related with worse pathogenic T, N, and M stages in LIHC patients (Fig. 6A). Following that, multivariate analysis revealed that the HSPA1A gene significantly predicted overall survival and progression-free interval, whereas the PPARGC1A gene significantly predicted overall survival, disease-specific survival, and progression-free interval (Fig. 6B–E) (Tables S13–S24). Even after controlling for confounding factors, these findings imply that the HSPA1A and PPARGC1A genes are independent predictive markers for LIHC patients.

### Expression of HSPA1A and PPARGC1A in the liver tissues of NASH mice and HCC patients

**Figure 7:**
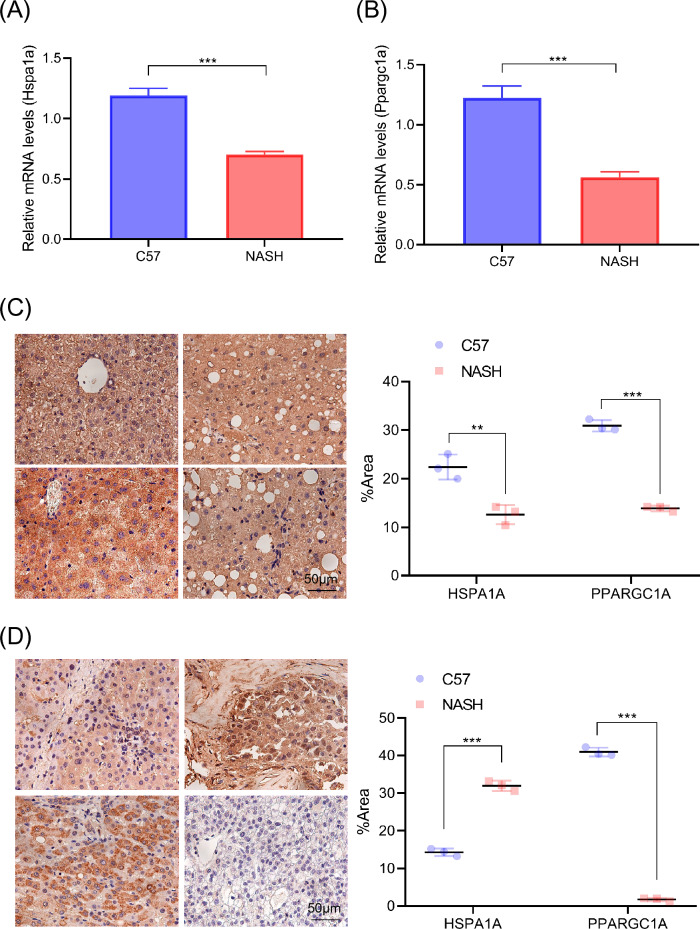
Identification of HSPA1A and PPARGC1A expression in liver tissues of NASH mice and HCC patients. Expression of Hspa1a (**A**) and Ppargc1a (**B**) at mRNA level in mouse liver tissue. (**C**) Hspa1a and Ppargc1a in mice liver tissue of immunohistochemical staining and quantitative. (**D**) Immunohistochemical staining and quantification of HSPA1A and PPARGC1A in human HCC tissues. ** *p* < 0.01; *** *p* < 0.001.

According to the results of the aforementioned study, we discovered that MAT1A and NR1I3 were not substantially connected with prognosis in the TCGA-LIHC cohort, indicating that HSPA1A and PPARGC1A may be crucial in the development of B[a]P-NASH-HCC. Using the human normal liver cell line LO2, we observed a significant decrease in HSPA1A mRNA expression and a significant increase in PPARGC1A mRNA expression following stimulation with B[a]P, when compared to normal cells (Figure S1A). Furthermore, upon stimulation with palmitic acid (PA), both genes exhibited a significant decrease in mRNA expression levels compared to normal cells (Figure S1B). We isolated RNA from the liver tissue of NASH model mice and carried out qPCR to determine if these two genes would be the essential genes for the therapy of NASH or HCC. The findings, which were in line with those of an examination of a human database, demonstrated that the genes for Hspa1a and Ppargc1a were down-expressed in the liver tissue of NASH animals (Fig. 7A,B). We subsequently conducted immunohistochemistry labeling on NASH mouse liver tissue sections and discovered that HSPA1A and PPARGC1A were down-regulated in Nash mouse liver tissues (Fig. 7C). To validate our findings, we did immunohistochemistry labeling on HCC patient tissue sections and discovered that HSPA1A was greatly overexpressed in the liver of HCC patients, but PPARGC1A expression was dramatically reduced (Fig. 7D). This implies that HSPA1A is a risk factor for HCC and that it is suppressed in the early stages. PPARGC1A, on the other hand, is a protective factor in HCC and is antagonistically overexpressed during the early stages.

## Discussion

In recent years, despite significant advancements in the identification and analysis of B[a]P and its detrimental effects on the liver, effectively preventing or reducing its irreversible hazardous impact on the development of hepatocellular carcinoma (HCC) remains elusive, resulting in unacceptably high mortality rates. Furthermore, studies focusing on comprehending their role in HCC have yielded limited clarity. Our objective was to gain novel insights into the molecular mechanisms underlying B[a]P-induced non-alcoholic steatohepatitis (NASH) and the progression of HCC through comprehensive bioinformatics analysis, with the aim of identifying potential targets for HCC diagnosis and treatment. The analysis identified HSPA1A and PPARGC1A as pivotal genes in the progression from B[a]P exposure to NASH and subsequent development of HCC. Although NR1I3 and MAT1A exhibited significant differential expression across the three GEO datasets, they did not demonstrate prognostic value within the TCGA-LIHC cohort.

HSPA1A, also known as heat shock protein 70 (HSP70), is a well-characterized member of the heat shock protein family^19^. These proteins are crucial in cellular stress responses, playing diverse roles in maintaining cellular homeostasis and ensuring cell survival under adverse conditions^20^. HSPA1A is involved in various cellular processes, including protein folding, protein trafficking, and protection against protein aggregation^21,22^. It also participates in DNA repair mechanisms, maintaining genomic stability and integrity^23–25^.

The observed significant decrease in HSPA1A expression in liver samples exposed to benzo[a]pyrene (B[a]P) and NASH suggests a potential protective response by hepatocytes to mitigate the harmful effects of B[a]P exposure and restore normal cellular functioning. This downregulation may reflect an adaptive mechanism by hepatocytes to reduce the burden of B[a]P-induced damage and promote cellular recovery. The reversible nature of NASH may contribute to this pattern, indicating a dynamic process of cellular adaptation and repair.

Interestingly, the upregulation of HSPA1A expression in liver cancer samples indicates its potential involvement in tumor progression. This finding suggests that HSPA1A may acquire new functions in the context of HCC development. HSPA1A can support the survival and proliferation of cancer cells by stabilizing oncogenic proteins or promoting the degradation of tumor suppressors^26^. Additionally, HSPA1A has been implicated in the evasion of immune surveillance, allowing cancer cells to escape immune recognition and destruction^27^. Therefore, it is possible that the decreased expression of HSPA1A represents a protective mechanism initiated by the body to cope with the continuous genetic and cellular changes associated with tumor formation. By downregulating HSPA1A, the body may attempt to limit the growth and progression of cancer cells.

Moving on to PPARGC1A, it encodes peroxisome proliferator-activated receptor gamma coactivator 1-alpha, a key regulator of cellular energy metabolism and mitochondrial biogenesis^28,29^. PPARGC1A interacts with various transcription factors to enhance the expression of genes involved in mitochondrial function, oxidative phosphorylation, and fatty acid oxidation^30,31^. It also plays a role in regulating adaptive thermogenesis and maintaining cellular redox balance^32,33^.

The observed increase in PPARGC1A expression in liver cells exposed to B[a]P suggests an initial protective response against the early risks associated with B[a]P exposure. This upregulation serves as a vital cellular defense mechanism aimed at enhancing mitochondrial biogenesis and energy metabolism. By increasing PPARGC1A expression, hepatocytes may attempt to counteract the detrimental effects of B[a]P and maintain cellular homeostasis. This adaptive response is likely aimed at mitigating the toxic effects of B[a]P, reducing the risk of DNA damage, and preventing the occurrence of mutations.

However, as NASH and HCC progress, there is a subsequent decrease in PPARGC1A expression. This downregulation of PPARGC1A is consistent with the metabolic dysregulation and oxidative stress observed in NASH and HCC^34^. These conditions are characterized by mitochondrial dysfunction, impaired energy metabolism, and increased levels of oxidative stress^35,36^. The decrease in PPARGC1A expression further exacerbates these pathological changes, contributing to the loss of mitochondrial integrity, impaired cellular bioenergetics, and disrupted redox homeostasis that drive disease progression^37^.

The diminishing effect on PPARGC1A expression in NASH and HCC is likely attributed to a combination of factors. Disease progression and genetic alterations accumulate over time, leading to a decline in PPARGC1A expression. As the disease advances, the mechanisms that initially upregulated PPARGC1A in response to B[a]P become less effective, resulting in a reduced ability to counteract the metabolic and oxidative stress-related challenges faced by liver cells. This decreased expression of PPARGC1A ultimately contributes to the worsening of mitochondrial dysfunction, impaired energy metabolism, and increased oxidative stress observed in NASH and HCC.

The high positive correlation between the expression of HSPA1A and PPARGC1A genes and the infiltration of nTreg cells, B cells, macrophages, and monocytes in TCGA-LIHC suggests a potential role for these genes in regulating immune cell infiltration within the tumor microenvironment of HCC. The presence of this correlation indicates that HSPA1A and PPARGC1A may be involved in creating an immunosuppressive microenvironment favorable for tumor growth and progression.

Interestingly, when comparing the ssGSEA scores of immune cells between HSPA1A high and low expression groups, as well as PPARGC1A high and low expression groups, immune B cells showed a significant correlation. This suggests that the expression levels of HSPA1A and PPARGC1A might have a specific impact on the behavior and function of immune B cells in the tumor microenvironment of HCC. B cells are primarily known for their role in producing antibodies, which can target and neutralize antigens associated with cancer cells^38^. Therefore, the specific influence of HSPA1A and PPARGC1A on immune B cells suggests a potential impact on the production of antibodies targeting HCC-related antigens. Manipulating the expression levels of these genes might have implications for enhancing anti-tumor antibody responses and improving the effectiveness of immune-mediated approaches against HCC. Additionally, B cells can act as antigen-presenting cells, carrying antigens from cancer cells to activate T cells and initiate an immune response^39^. By modulating the behavior of B cells, HSPA1A and PPARGC1A might affect the antigen presentation process, influencing the activation and function of tumor-specific T cells^40^. This suggests that manipulating the expression of these genes could potentially enhance the ability of B cells to prime and activate anti-tumor T cell responses. Furthermore, B cells can also exert regulatory functions by interacting with other immune cells and modulating their activity. Through their production of cytokines and interaction with immune checkpoints, B cells can regulate the balance between pro-inflammatory and suppressive immune responses. The specific influence of HSPA1A and PPARGC1A on immune B cells implies that these genes might impact the regulatory functions of B cells within the HCC tumor microenvironment. This has implications for understanding the complex interplay between different immune cell populations in HCC and highlights potential therapeutic targets for modulating immune regulation in the tumor microenvironment.

On TCGA-LIHC patients, we performed multivariate and univariate cox regression analysis and discovered that the HSPA1A gene significantly predicted overall survival and progression-free interval, whereas the PPARGC1A gene significantly predicted overall survival, disease-specific survival, and progression-free interval. Consistent with the above results, Hspa1a and Ppargc1a were found to be down-regulated in the liver tissues of NASH mice. In order to further validate the importance of two genes for HCC, we on human liver biopsy in patients with immune dyeing, found HSPA1A expressed in human liver cancer samples was significantly higher, while PPARGC1A significantly lower expression, and consistent with early results^41^.

Our study has certain limitations. While our bioinformatics analysis provided valuable insights into the molecular mechanisms underlying B[a]P-induced NASH and HCC, it is important to note that the shared genes between B[a]P exposure, NASH, and HCC do not necessarily imply a direct causal relationship among them. Additionally, we recognize the need for experimental validation to support our predicted protein–protein interactions. This validation would further strengthen the reliability and robustness of our findings. Moreover, our study was limited by the sample size, and our predictions need validation in a larger cohort. These insights may pave the way for the development of novel strategies to prevent or treat HCC associated with B[a]P exposure, ultimately reducing the burden of liver cancer globally.

In conclusion, our study unravels the intricate connections between B[a]P exposure, NASH, and HCC. Through comprehensive bioinformatics analysis and experimental studies, we identified key genes, particularly HSPA1A and PPARGC1A, associated with the B[a]P-NASH-HCC pathway. These findings provide crucial insights into the regulatory mechanisms underlying B[a]P-induced hepatotoxicity and shed light on the pathogenesis of B[a]P-associated NASH and HCC. Enhancing our understanding of these regulatory roles may pave the way for the development of targeted therapies, ultimately improving patient outcomes. Overall, our research not only enhances our understanding of the detrimental effects of B[a]P on liver health but also opens avenues for potential interventions to prevent or treat NASH and HCC in individuals exposed to B[a]P.

## Methods

### Animals

The Nanjing Model Animal Center (Nanjing, China) provided male C57BL/6 J mice (seven weeks old). Mice were randomly separated into two groups, one given a normal diet (ND) and the other a high fat diet (HFD) for eight weeks (n = 6 per group) to simulated NASH model in mice^42^. After 8 weeks, all mice were sedated with isoflurane and killed through cervical dislocation by followed the American Veterinary Medical Association​(AVMA) Guidelines for the Euthanasia of Animals (2020). Directive 2010/63/EU, Commission Implementing Decision (EU) 2020/569, Recommendation 2007/526/EC, and the 1991 International Guidelines for Ethical Review of Epidemiological Studies were all followed in all animal studies. We affirm that this study has been reported following the ARRIVE guidelines (https://arriveguidelines.org).

### Sample collection

Human liver cancer tissue samples were collected from patients diagnosed with liver cancer, while normal tissue samples were obtained from individuals without any liver diseases. All samples were collected following proper ethical guidelines and with the informed consent of the patients/donors.

### Cell culture and treatment

The human normal liver cell line LO2 was obtained from the Cell Bank at the Chinese Academy of Sciences. LO2 cells were cultured in Dulbecco's modified Eagle medium (Life Technologies Corporation, USA) supplemented with 10% fetal bovine serum. To induce cellular effects, LO2 cells were treated with 25 μM B[a]P (Sigma, B1760, ≥ 96% HPLC) dissolved in dimethyl sulfoxide (DMSO) or 300 μM palmitic acid (Sigma) for 24 h.

### Histology

In order to conduct histological examinations, liver samples were first fixed in 4% paraformaldehyde for 48 h before being dried, embedded in paraffin, and then sectioned. The coloring of the tissue slices was achieved using immunohistochemical staining.

### RNA isolation and qPCR

TRIzol reagent (Invitrogen) was used for the RNA extraction process. 1 g of total RNA was reverse-transcribed into cDNA using a PrimeScriptTM RT reagent Kit with gDNA Eraser from Takara in Beijing, China. Quantitative real-time PCR was carried out using a CFX96 TouchTM Real-Time PCR Detection System (Hercules, CA, USA) utilizing TB Green® Fast quantitative polymerase chain reaction (qPCR) Mix (Takara) and particular primers (Ribobio).

### Data source and identification of DEGs

From the GEO database, three microarray gene expression datasets (GSE127791, GSE164760, and GSE146049) pertaining to liver injury were chosen. Human terminally differentiated hepatic HepaRG cells were treated with 1 μM B[a]P in the GSE127791 dataset, and the DEGs were identified. The GSE164760 dataset contained gene expression data from 74 patients with NASH and 6 healthy individuals in order to investigate the altered genes implicated in the progression of human NASH. In the GSE146049 dataset, 5 liver cancer tumorous tissues were compared to 5 samples from non-tumor liver to identify the DEGs. Both male and female patients were included in all data sets. The difference analysis of all data sets was performed using R Project for Statistical Computing (RRID:SCR_001905), LIMMA (RRID:SCR_010943) and DESeq2 (RRID:SCR_015687) were used to standardization and differential analysis. The volcano plots and heatmaps of the DEGs in each dataset were constructed using the SRplot web tool (http://www.bioinformatics.com.cn/srplot), with a statistically significant cutoff value of |log2(FC)|> 0.5 and *p* < 0.05. We discovered 39 genes by overlapping the DEGs from these three GEO datasets.

### Gene ontology (GO) and kyoto encyclopedia of genes and genomes (KEGG) analysis

The combined set of 39 DEGs from the three previously mentioned GEO datasets was analyzed for GO enrichment and KEGG pathway enrichment using the ShinyGO 0.77 online tool (RRID:SCR_019213), which provides a user-friendly and visually appealing interface for enrichment analysis. Top ten pathways were chosen for both the GO and KEGG studies based on their relevance^43,44^. To identify the enriched pathways, a false discovery rate (FDR) value of less than 0.05 was used as the cut-off criterion.

### Protein–protein interaction (PPI) and hub gene discovery

To integrate biomolecular interaction networks with high-throughput expression data and other molecular states, we obtained protein and functional interaction networks for the 39 combined DEGs from the STRING database (RRID:SCR_005223, https://string-db.org/) and visualized them using Cytoscape^45^. To investigate the hub gene network, we utilized the CytoHubb Plugin for Cytoscape (RRID:SCR_003032). This plugin offers a variety of techniques for identifying critical nodes in biological networks and their connections to other genes. In our study, the top 10 hub genes were identified by employing diverse algorithms (closeness, MNC, EPC, and betweenness ranking methods) to identify hub genes. The resulting network colored the hub nodes according to their relevance, with red representing the greatest score and yellow representing the lowest. We identified HSPA1A, NR1I3, PPARGC1A, and MAT1A as the common interacting hub genes after completing a Venn diagram analysis on the top 10 hub genes from each ranking method. In addition, network analysis was used to look into the protein–protein interactions involving these hub genes. The online program ShinyGO 0.77 was used to demonstrate the enrichment of the interactor network using Gene Ontology biological process pathway (GO-BP) and KEGG pathway.

### Gene set enrichment analysis (GSEA)

We separated the TCGA-LIHC samples into two groups based on the average gene expression value to find enriched gene sets based on the highly expressed group of HSPA1A, NR1I3, PPARGC1A, and MAT1A genes. We used GSEA software (RRID:SCR_005724) to run 1000 permutations of the KEGG pathways (c2.cp.kegg.v2023.1) and GO-BP gene sets (c5.go.bp.v2023.1) gene sets to determine the enrichment score (ES). Based on the following criteria, the ten gene sets with the highest statistical significance were chosen: |NES|> 1, NOM *p*-value < 0.05, and FDR q-value < 0.25, indicating statistical significance. In addition, we used the Kaplan–Meier method and Cox regression analysis to determine the effect of HSPA1A, NR1I3, PPARGC1A, and MAT1A gene expression on prognosis. Variables with a *p*-value less than 0.1 in the univariate Cox regression were included in the multivariate Cox regression in the Cox regression study. A *p*-value less than 0.05 was considered statistically significant.

### Statistical analysis

An independent Student's t-test in GraphPad Prism (version 8.0.1, RRID:SCR_002798) was used to analyze the difference in HSPA1A, NR1I3, PPARGC1A, and MAT1A gene expression between distinct sample groups (Benzopyrene vs. control, NASH vs. healthy, tumors vs. normal). A chi-square test was used in R software (version 4.2.1) to examine the relationship between high and low expression of HSPA1A, NR1I3, PPARGC1A, and MAT1A genes and clinical phenotypic data in the TCGA-LIHC dataset. Statistical significance was set at *p* < 0.05, and the level of significance was indicated as follows: * *p* < 0.05, ** *p* < 0.01, *** *p* < 0.001, ****p < 0.0001.

### Statement

Prior to sample collection, all patients/donors were provided with detailed information about the study objectives, procedures, and potential risks. Informed consent was obtained from each participant, ensuring their voluntary participation in the study. Human experiments complied with the Ethical guidelines of The Code of Ethics of the World Medical Association (Declaration of Helsinki). The research protocol was reviewed and approved by the General Hospital of Northern Theater Command before the commencement of the study. The study had been performed in accordance with the Declaration of Helsinki.

### Supplementary Information

Supplementary Information.

## Data Availability

The data used to support our results are available at the GEO (https://www.ncbi.nlm.nih.gov/geo/ accessed on 1st June 2023), TCGA (https://portal.gdc.cancer.gov/ accessed on 14 June 2023)**.**

## Abbreviations

B[a]P: Benzopyrene
NASH: Nonalcoholic steatohepatitis
HCC: Hepatocellular carcinoma
DEG: Differentially expressed genes
PPI: Protein–Protein interaction
GEO: Gene Expression Omnibus
TCGA: The Cancer Genome Atlas
KEGG: Kyoto encyclopedia of genes and genomes
GO-BP: Gene ontology biological process
HSPA1A: Heat Shock Protein Family A (Hsp70) Member 1A
NR1I3: Nuclear Receptor Subfamily 1 Group I Member 3
PPARGC1A: PPARG Coactivator 1 Alpha
MAT1A: Methionine Adenosyltransferase 1A
