# Supplementary material for: Unraveling the molecular links between benzopyrene exposure, NASH, and HCC: an integrated bioinformatics and experimental study
Source: Sci Rep. 2023 Nov 22;13:20520. doi: 10.1038/s41598-023-46440-1 (PMC10665343; doi:10.1038/s41598-023-46440-1)
Supplement: Supplementary file 1 — Supplementary Information. [file 41598_2023_46440_MOESM1_ESM.ppt]

## Slide 1
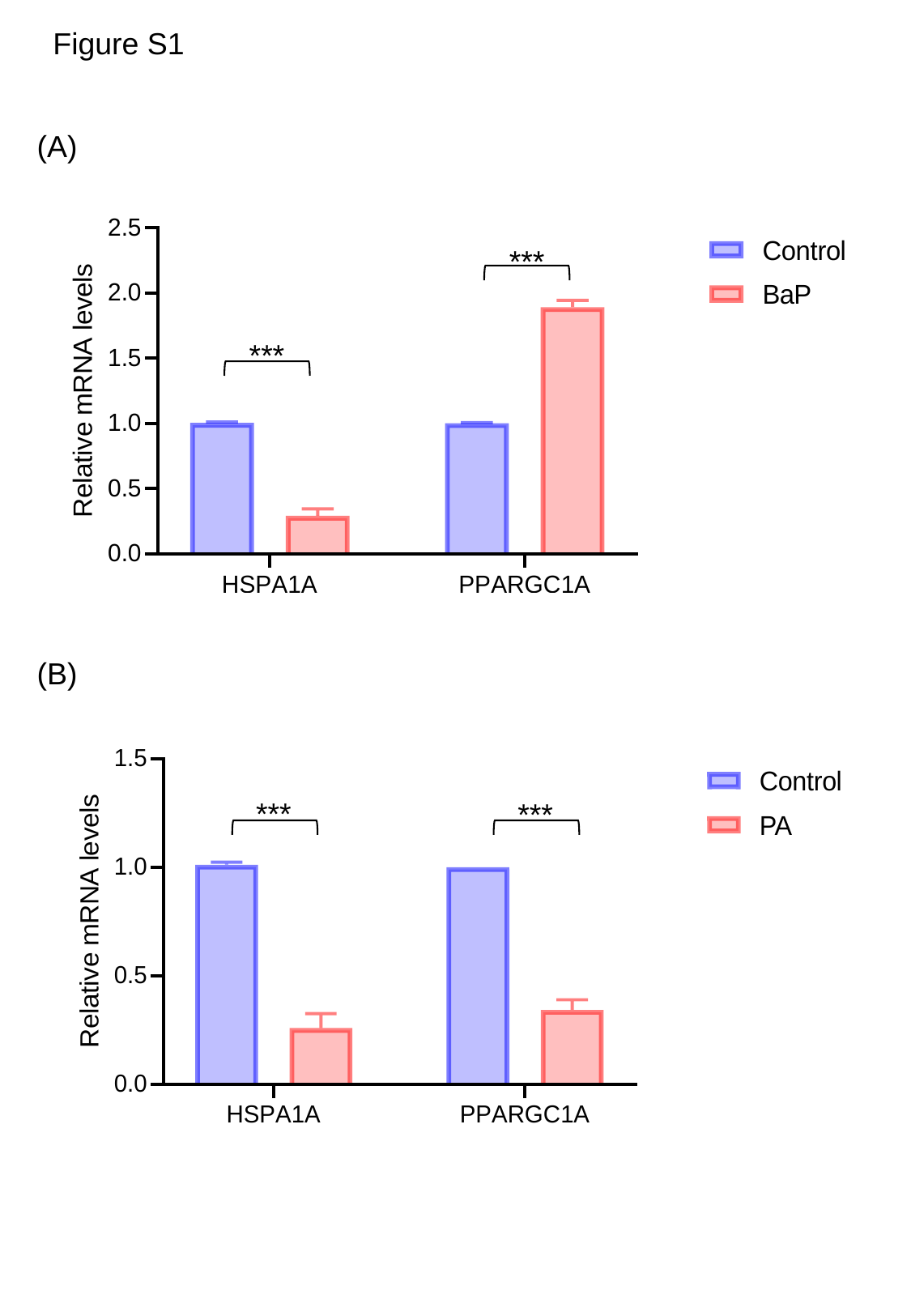

Figure S1
(A)
***
***
(B)
***
***

## Slide 2
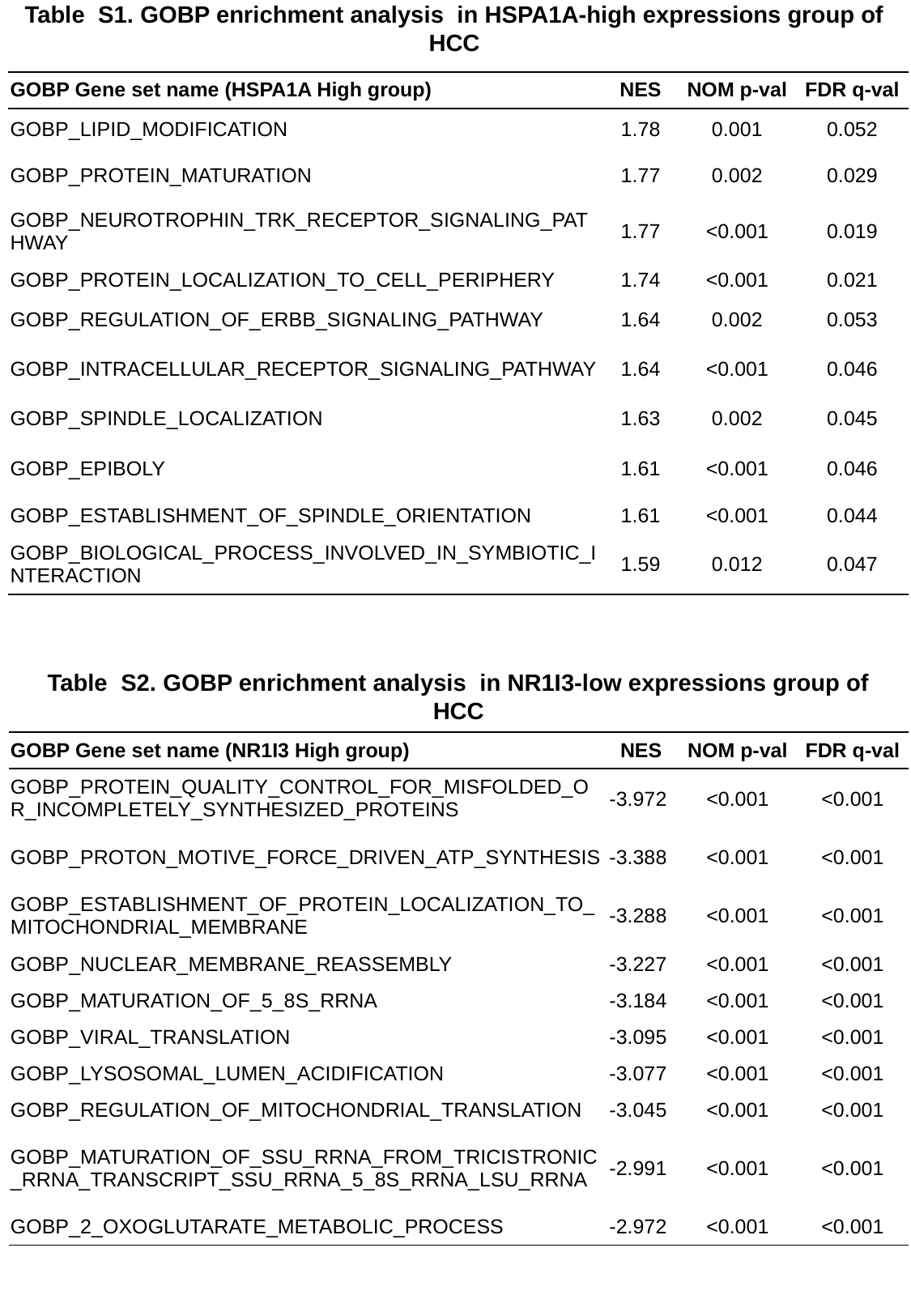

Table S1. GOBP enrichment analysis in HSPA1A-high expressions group of HCC
| GOBP Gene set name (HSPA1A High group) | NES | NOM p-val | FDR q-val |
| --- | --- | --- | --- |
| GOBP\_LIPID\_MODIFICATION | 1.78 | 0.001 | 0.052 |
| GOBP\_PROTEIN\_MATURATION | 1.77 | 0.002 | 0.029 |
| GOBP\_NEUROTROPHIN\_TRK\_RECEPTOR\_SIGNALING\_PATHWAY | 1.77 | <0.001 | 0.019 |
| GOBP\_PROTEIN\_LOCALIZATION\_TO\_CELL\_PERIPHERY | 1.74 | <0.001 | 0.021 |
| GOBP\_REGULATION\_OF\_ERBB\_SIGNALING\_PATHWAY | 1.64 | 0.002 | 0.053 |
| GOBP\_INTRACELLULAR\_RECEPTOR\_SIGNALING\_PATHWAY | 1.64 | <0.001 | 0.046 |
| GOBP\_SPINDLE\_LOCALIZATION | 1.63 | 0.002 | 0.045 |
| GOBP\_EPIBOLY | 1.61 | <0.001 | 0.046 |
| GOBP\_ESTABLISHMENT\_OF\_SPINDLE\_ORIENTATION | 1.61 | <0.001 | 0.044 |
| GOBP\_BIOLOGICAL\_PROCESS\_INVOLVED\_IN\_SYMBIOTIC\_INTERACTION | 1.59 | 0.012 | 0.047 |
Table S2. GOBP enrichment analysis in NR1I3-low expressions group of HCC
| GOBP Gene set name (NR1I3 High group) | NES | NOM p-val | FDR q-val |
| --- | --- | --- | --- |
| GOBP\_PROTEIN\_QUALITY\_CONTROL\_FOR\_MISFOLDED\_OR\_INCOMPLETELY\_SYNTHESIZED\_PROTEINS | -3.972 | <0.001 | <0.001 |
| GOBP\_PROTON\_MOTIVE\_FORCE\_DRIVEN\_ATP\_SYNTHESIS | -3.388 | <0.001 | <0.001 |
| GOBP\_ESTABLISHMENT\_OF\_PROTEIN\_LOCALIZATION\_TO\_MITOCHONDRIAL\_MEMBRANE | -3.288 | <0.001 | <0.001 |
| GOBP\_NUCLEAR\_MEMBRANE\_REASSEMBLY | -3.227 | <0.001 | <0.001 |
| GOBP\_MATURATION\_OF\_5\_8S\_RRNA | -3.184 | <0.001 | <0.001 |
| GOBP\_VIRAL\_TRANSLATION | -3.095 | <0.001 | <0.001 |
| GOBP\_LYSOSOMAL\_LUMEN\_ACIDIFICATION | -3.077 | <0.001 | <0.001 |
| GOBP\_REGULATION\_OF\_MITOCHONDRIAL\_TRANSLATION | -3.045 | <0.001 | <0.001 |
| GOBP\_MATURATION\_OF\_SSU\_RRNA\_FROM\_TRICISTRONIC\_RRNA\_TRANSCRIPT\_SSU\_RRNA\_5\_8S\_RRNA\_LSU\_RRNA | -2.991 | <0.001 | <0.001 |
| GOBP\_2\_OXOGLUTARATE\_METABOLIC\_PROCESS | -2.972 | <0.001 | <0.001 |

## Slide 3
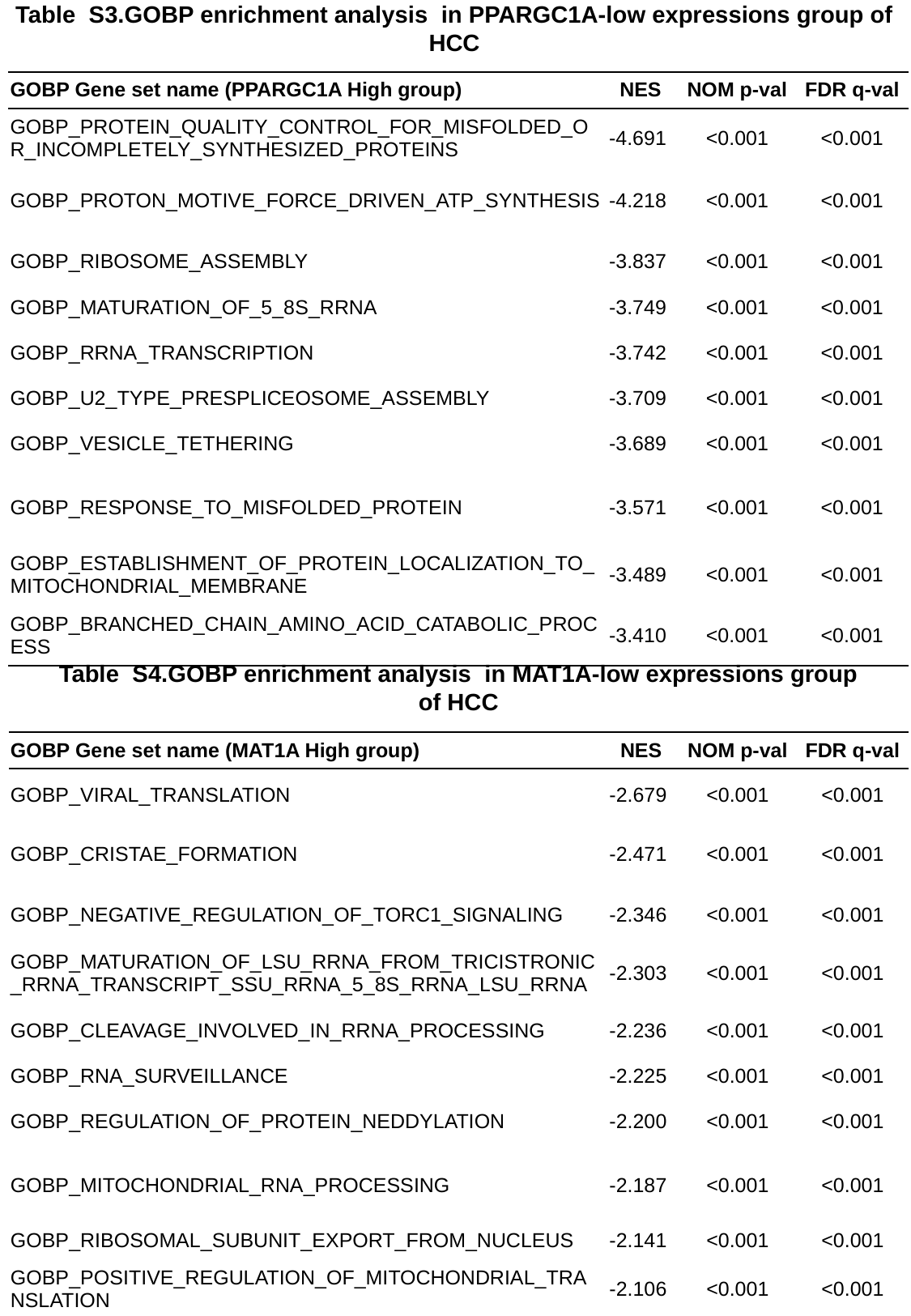

Table S3.GOBP enrichment analysis in PPARGC1A-low expressions group of HCC
| GOBP Gene set name (PPARGC1A High group) | NES | NOM p-val | FDR q-val |
| --- | --- | --- | --- |
| GOBP\_PROTEIN\_QUALITY\_CONTROL\_FOR\_MISFOLDED\_OR\_INCOMPLETELY\_SYNTHESIZED\_PROTEINS | -4.691 | <0.001 | <0.001 |
| GOBP\_PROTON\_MOTIVE\_FORCE\_DRIVEN\_ATP\_SYNTHESIS | -4.218 | <0.001 | <0.001 |
| GOBP\_RIBOSOME\_ASSEMBLY | -3.837 | <0.001 | <0.001 |
| GOBP\_MATURATION\_OF\_5\_8S\_RRNA | -3.749 | <0.001 | <0.001 |
| GOBP\_RRNA\_TRANSCRIPTION | -3.742 | <0.001 | <0.001 |
| GOBP\_U2\_TYPE\_PRESPLICEOSOME\_ASSEMBLY | -3.709 | <0.001 | <0.001 |
| GOBP\_VESICLE\_TETHERING | -3.689 | <0.001 | <0.001 |
| GOBP\_RESPONSE\_TO\_MISFOLDED\_PROTEIN | -3.571 | <0.001 | <0.001 |
| GOBP\_ESTABLISHMENT\_OF\_PROTEIN\_LOCALIZATION\_TO\_MITOCHONDRIAL\_MEMBRANE | -3.489 | <0.001 | <0.001 |
| GOBP\_BRANCHED\_CHAIN\_AMINO\_ACID\_CATABOLIC\_PROCESS | -3.410 | <0.001 | <0.001 |
Table S4.GOBP enrichment analysis in MAT1A-low expressions group of HCC
| GOBP Gene set name (MAT1A High group) | NES | NOM p-val | FDR q-val |
| --- | --- | --- | --- |
| GOBP\_VIRAL\_TRANSLATION | -2.679 | <0.001 | <0.001 |
| GOBP\_CRISTAE\_FORMATION | -2.471 | <0.001 | <0.001 |
| GOBP\_NEGATIVE\_REGULATION\_OF\_TORC1\_SIGNALING | -2.346 | <0.001 | <0.001 |
| GOBP\_MATURATION\_OF\_LSU\_RRNA\_FROM\_TRICISTRONIC\_RRNA\_TRANSCRIPT\_SSU\_RRNA\_5\_8S\_RRNA\_LSU\_RRNA | -2.303 | <0.001 | <0.001 |
| GOBP\_CLEAVAGE\_INVOLVED\_IN\_RRNA\_PROCESSING | -2.236 | <0.001 | <0.001 |
| GOBP\_RNA\_SURVEILLANCE | -2.225 | <0.001 | <0.001 |
| GOBP\_REGULATION\_OF\_PROTEIN\_NEDDYLATION | -2.200 | <0.001 | <0.001 |
| GOBP\_MITOCHONDRIAL\_RNA\_PROCESSING | -2.187 | <0.001 | <0.001 |
| GOBP\_RIBOSOMAL\_SUBUNIT\_EXPORT\_FROM\_NUCLEUS | -2.141 | <0.001 | <0.001 |
| GOBP\_POSITIVE\_REGULATION\_OF\_MITOCHONDRIAL\_TRANSLATION | -2.106 | <0.001 | <0.001 |

## Slide 4
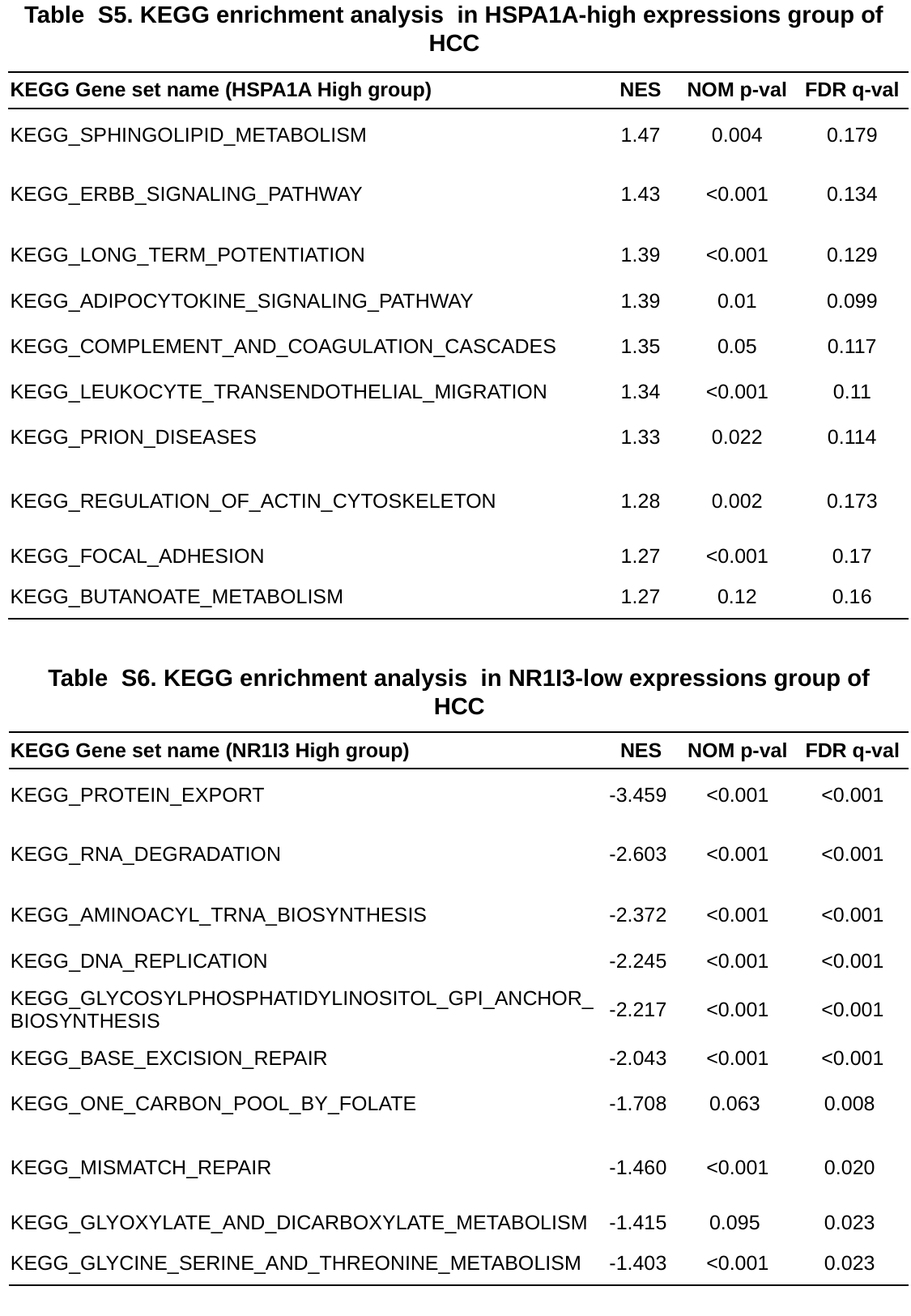

Table S5. KEGG enrichment analysis in HSPA1A-high expressions group of HCC
| KEGG Gene set name (HSPA1A High group) | NES | NOM p-val | FDR q-val |
| --- | --- | --- | --- |
| KEGG\_SPHINGOLIPID\_METABOLISM | 1.47 | 0.004 | 0.179 |
| KEGG\_ERBB\_SIGNALING\_PATHWAY | 1.43 | <0.001 | 0.134 |
| KEGG\_LONG\_TERM\_POTENTIATION | 1.39 | <0.001 | 0.129 |
| KEGG\_ADIPOCYTOKINE\_SIGNALING\_PATHWAY | 1.39 | 0.01 | 0.099 |
| KEGG\_COMPLEMENT\_AND\_COAGULATION\_CASCADES | 1.35 | 0.05 | 0.117 |
| KEGG\_LEUKOCYTE\_TRANSENDOTHELIAL\_MIGRATION | 1.34 | <0.001 | 0.11 |
| KEGG\_PRION\_DISEASES | 1.33 | 0.022 | 0.114 |
| KEGG\_REGULATION\_OF\_ACTIN\_CYTOSKELETON | 1.28 | 0.002 | 0.173 |
| KEGG\_FOCAL\_ADHESION | 1.27 | <0.001 | 0.17 |
| KEGG\_BUTANOATE\_METABOLISM | 1.27 | 0.12 | 0.16 |
Table S6. KEGG enrichment analysis in NR1I3-low expressions group of HCC
| KEGG Gene set name (NR1I3 High group) | NES | NOM p-val | FDR q-val |
| --- | --- | --- | --- |
| KEGG\_PROTEIN\_EXPORT | -3.459 | <0.001 | <0.001 |
| KEGG\_RNA\_DEGRADATION | -2.603 | <0.001 | <0.001 |
| KEGG\_AMINOACYL\_TRNA\_BIOSYNTHESIS | -2.372 | <0.001 | <0.001 |
| KEGG\_DNA\_REPLICATION | -2.245 | <0.001 | <0.001 |
| KEGG\_GLYCOSYLPHOSPHATIDYLINOSITOL\_GPI\_ANCHOR\_BIOSYNTHESIS | -2.217 | <0.001 | <0.001 |
| KEGG\_BASE\_EXCISION\_REPAIR | -2.043 | <0.001 | <0.001 |
| KEGG\_ONE\_CARBON\_POOL\_BY\_FOLATE | -1.708 | 0.063 | 0.008 |
| KEGG\_MISMATCH\_REPAIR | -1.460 | <0.001 | 0.020 |
| KEGG\_GLYOXYLATE\_AND\_DICARBOXYLATE\_METABOLISM | -1.415 | 0.095 | 0.023 |
| KEGG\_GLYCINE\_SERINE\_AND\_THREONINE\_METABOLISM | -1.403 | <0.001 | 0.023 |

## Slide 5
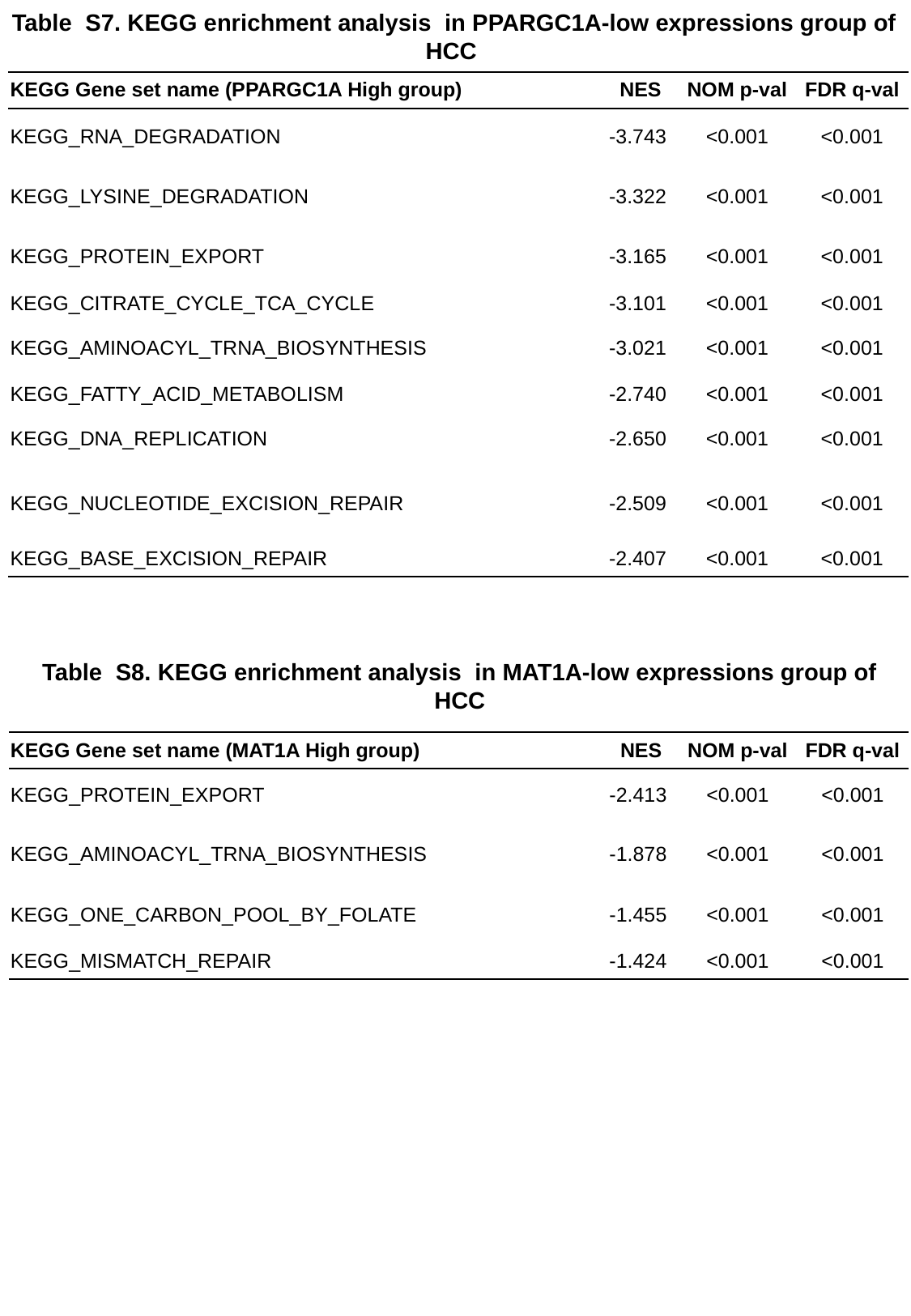

Table S7. KEGG enrichment analysis in PPARGC1A-low expressions group of HCC
| KEGG Gene set name (PPARGC1A High group) | NES | NOM p-val | FDR q-val |
| --- | --- | --- | --- |
| KEGG\_RNA\_DEGRADATION | -3.743 | <0.001 | <0.001 |
| KEGG\_LYSINE\_DEGRADATION | -3.322 | <0.001 | <0.001 |
| KEGG\_PROTEIN\_EXPORT | -3.165 | <0.001 | <0.001 |
| KEGG\_CITRATE\_CYCLE\_TCA\_CYCLE | -3.101 | <0.001 | <0.001 |
| KEGG\_AMINOACYL\_TRNA\_BIOSYNTHESIS | -3.021 | <0.001 | <0.001 |
| KEGG\_FATTY\_ACID\_METABOLISM | -2.740 | <0.001 | <0.001 |
| KEGG\_DNA\_REPLICATION | -2.650 | <0.001 | <0.001 |
| KEGG\_NUCLEOTIDE\_EXCISION\_REPAIR | -2.509 | <0.001 | <0.001 |
| KEGG\_BASE\_EXCISION\_REPAIR | -2.407 | <0.001 | <0.001 |
Table S8. KEGG enrichment analysis in MAT1A-low expressions group of HCC
| KEGG Gene set name (MAT1A High group) | NES | NOM p-val | FDR q-val |
| --- | --- | --- | --- |
| KEGG\_PROTEIN\_EXPORT | -2.413 | <0.001 | <0.001 |
| KEGG\_AMINOACYL\_TRNA\_BIOSYNTHESIS | -1.878 | <0.001 | <0.001 |
| KEGG\_ONE\_CARBON\_POOL\_BY\_FOLATE | -1.455 | <0.001 | <0.001 |
| KEGG\_MISMATCH\_REPAIR | -1.424 | <0.001 | <0.001 |

## Slide 6
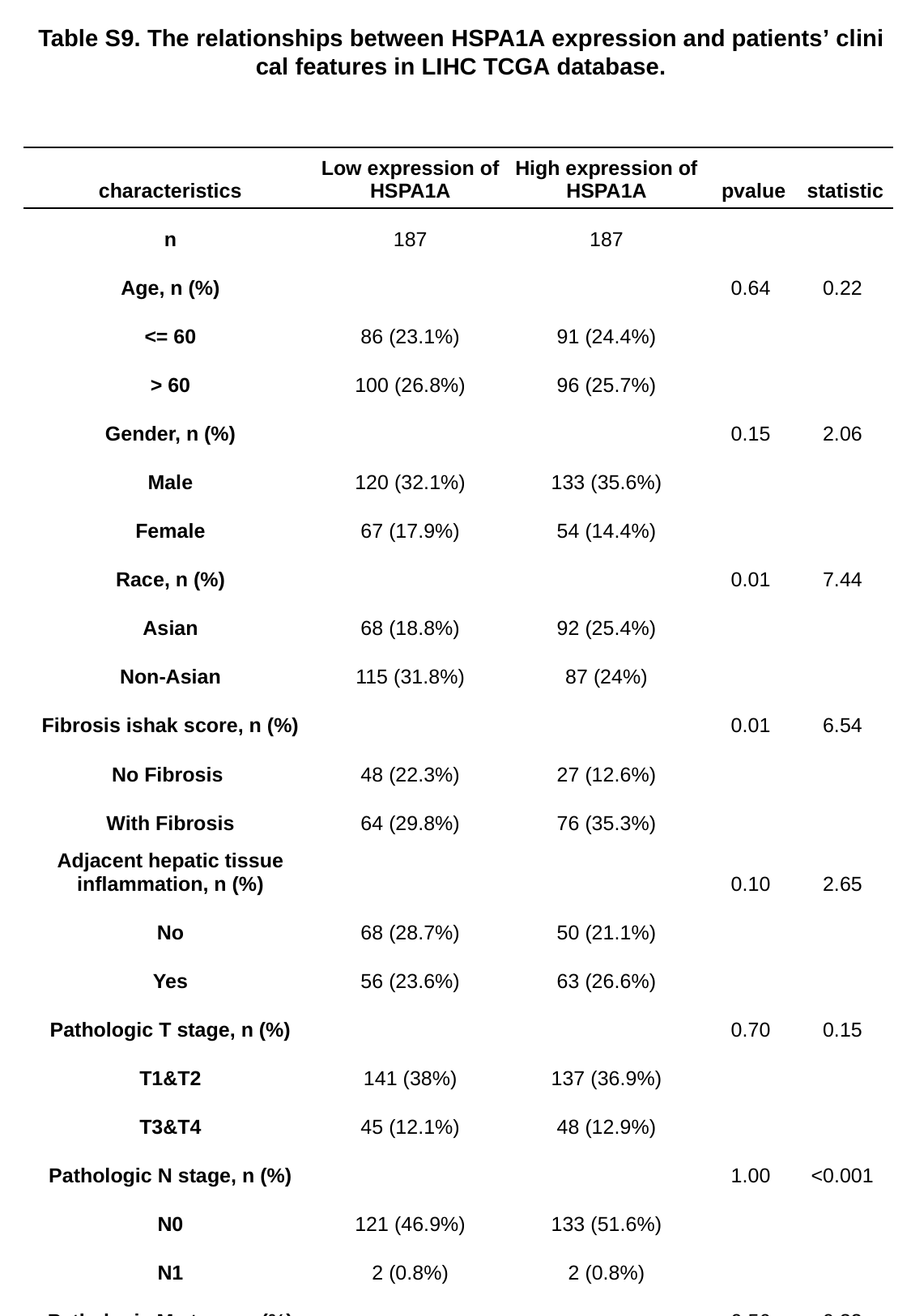

Table S9. The relationships between HSPA1A expression and patients’ clinical features in LIHC TCGA database.
| characteristics | Low expression of HSPA1A | High expression of HSPA1A | pvalue | statistic |
| --- | --- | --- | --- | --- |
| n | 187 | 187 | | |
| Age, n (%) | | | 0.64 | 0.22 |
| <= 60 | 86 (23.1%) | 91 (24.4%) | | |
| > 60 | 100 (26.8%) | 96 (25.7%) | | |
| Gender, n (%) | | | 0.15 | 2.06 |
| Male | 120 (32.1%) | 133 (35.6%) | | |
| Female | 67 (17.9%) | 54 (14.4%) | | |
| Race, n (%) | | | 0.01 | 7.44 |
| Asian | 68 (18.8%) | 92 (25.4%) | | |
| Non-Asian | 115 (31.8%) | 87 (24%) | | |
| Fibrosis ishak score, n (%) | | | 0.01 | 6.54 |
| No Fibrosis | 48 (22.3%) | 27 (12.6%) | | |
| With Fibrosis | 64 (29.8%) | 76 (35.3%) | | |
| Adjacent hepatic tissue inflammation, n (%) | | | 0.10 | 2.65 |
| No | 68 (28.7%) | 50 (21.1%) | | |
| Yes | 56 (23.6%) | 63 (26.6%) | | |
| Pathologic T stage, n (%) | | | 0.70 | 0.15 |
| T1&T2 | 141 (38%) | 137 (36.9%) | | |
| T3&T4 | 45 (12.1%) | 48 (12.9%) | | |
| Pathologic N stage, n (%) | | | 1.00 | <0.001 |
| N0 | 121 (46.9%) | 133 (51.6%) | | |
| N1 | 2 (0.8%) | 2 (0.8%) | | |
| Pathologic M stage, n (%) | | | 0.56 | 0.33 |
| M0 | 128 (47.1%) | 140 (51.5%) | | |
| M1 | 3 (1.1%) | 1 (0.4%) | | |

## Slide 7
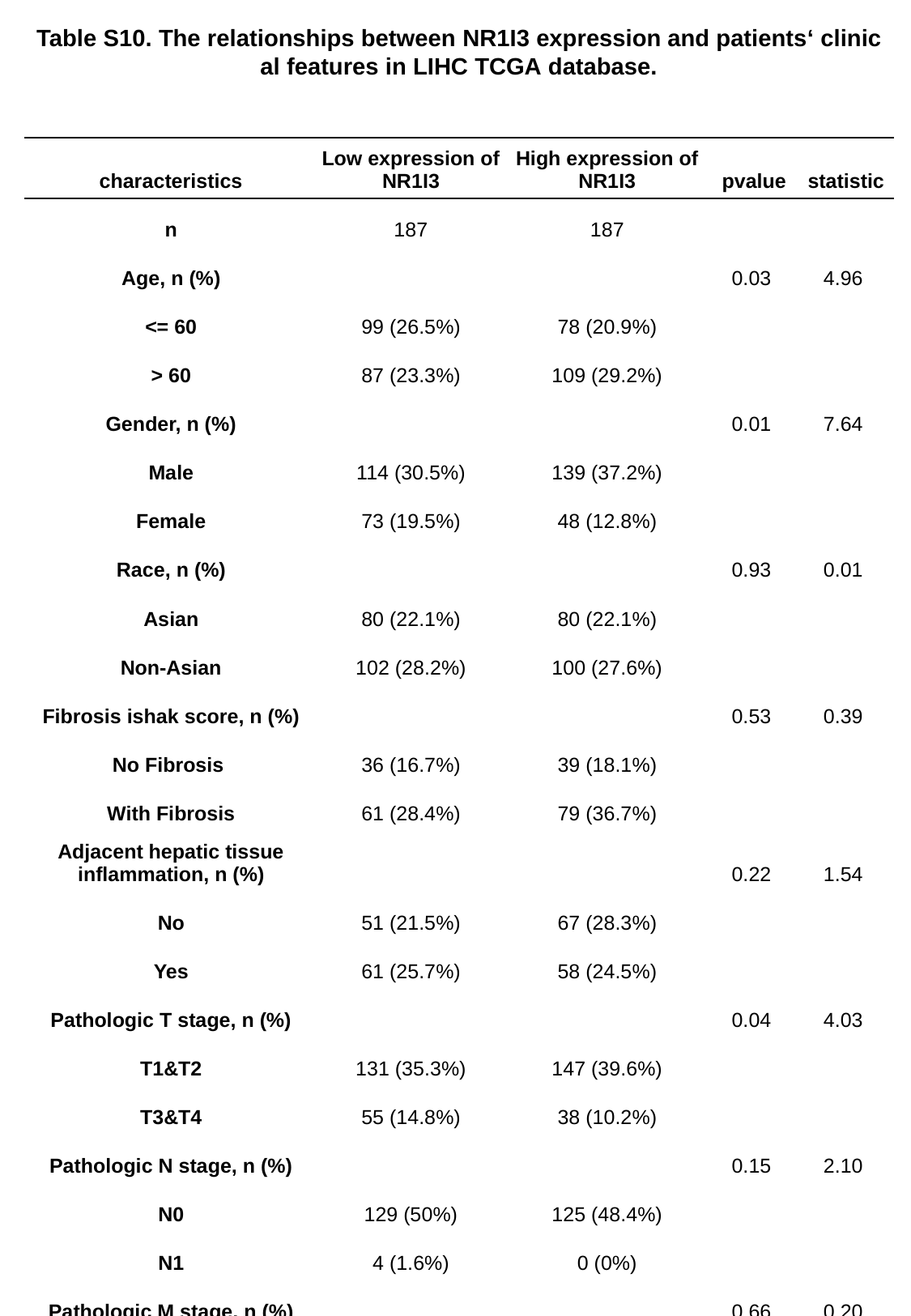

Table S10. The relationships between NR1I3 expression and patients‘ clinical features in LIHC TCGA database.
| characteristics | Low expression of NR1I3 | High expression of NR1I3 | pvalue | statistic |
| --- | --- | --- | --- | --- |
| n | 187 | 187 | | |
| Age, n (%) | | | 0.03 | 4.96 |
| <= 60 | 99 (26.5%) | 78 (20.9%) | | |
| > 60 | 87 (23.3%) | 109 (29.2%) | | |
| Gender, n (%) | | | 0.01 | 7.64 |
| Male | 114 (30.5%) | 139 (37.2%) | | |
| Female | 73 (19.5%) | 48 (12.8%) | | |
| Race, n (%) | | | 0.93 | 0.01 |
| Asian | 80 (22.1%) | 80 (22.1%) | | |
| Non-Asian | 102 (28.2%) | 100 (27.6%) | | |
| Fibrosis ishak score, n (%) | | | 0.53 | 0.39 |
| No Fibrosis | 36 (16.7%) | 39 (18.1%) | | |
| With Fibrosis | 61 (28.4%) | 79 (36.7%) | | |
| Adjacent hepatic tissue inflammation, n (%) | | | 0.22 | 1.54 |
| No | 51 (21.5%) | 67 (28.3%) | | |
| Yes | 61 (25.7%) | 58 (24.5%) | | |
| Pathologic T stage, n (%) | | | 0.04 | 4.03 |
| T1&T2 | 131 (35.3%) | 147 (39.6%) | | |
| T3&T4 | 55 (14.8%) | 38 (10.2%) | | |
| Pathologic N stage, n (%) | | | 0.15 | 2.10 |
| N0 | 129 (50%) | 125 (48.4%) | | |
| N1 | 4 (1.6%) | 0 (0%) | | |
| Pathologic M stage, n (%) | | | 0.66 | 0.20 |
| M0 | 137 (50.4%) | 131 (48.2%) | | |
| M1 | 3 (1.1%) | 1 (0.4%) | | |

## Slide 8
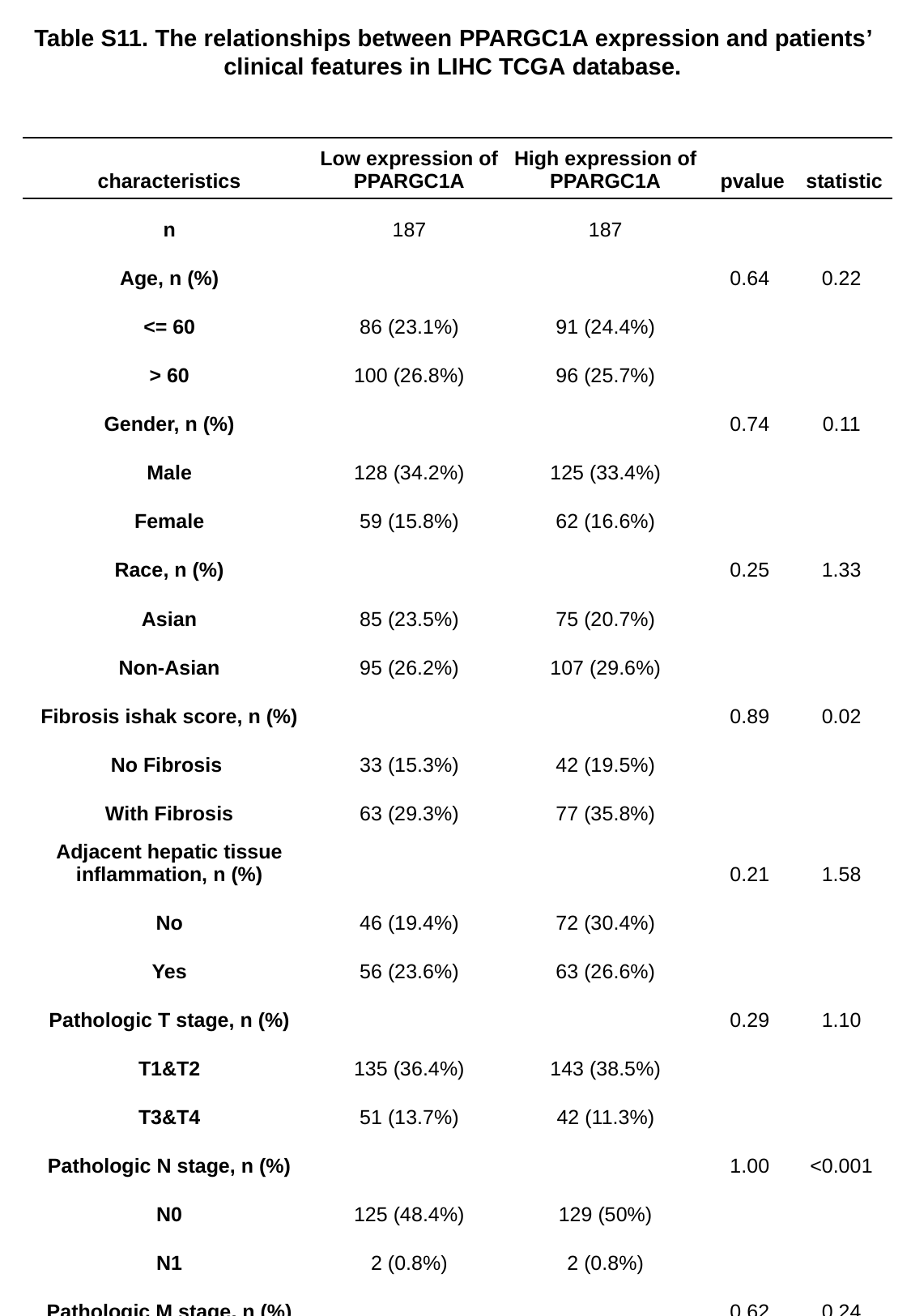

Table S11. The relationships between PPARGC1A expression and patients’ clinical features in LIHC TCGA database.
| characteristics | Low expression of PPARGC1A | High expression of PPARGC1A | pvalue | statistic |
| --- | --- | --- | --- | --- |
| n | 187 | 187 | | |
| Age, n (%) | | | 0.64 | 0.22 |
| <= 60 | 86 (23.1%) | 91 (24.4%) | | |
| > 60 | 100 (26.8%) | 96 (25.7%) | | |
| Gender, n (%) | | | 0.74 | 0.11 |
| Male | 128 (34.2%) | 125 (33.4%) | | |
| Female | 59 (15.8%) | 62 (16.6%) | | |
| Race, n (%) | | | 0.25 | 1.33 |
| Asian | 85 (23.5%) | 75 (20.7%) | | |
| Non-Asian | 95 (26.2%) | 107 (29.6%) | | |
| Fibrosis ishak score, n (%) | | | 0.89 | 0.02 |
| No Fibrosis | 33 (15.3%) | 42 (19.5%) | | |
| With Fibrosis | 63 (29.3%) | 77 (35.8%) | | |
| Adjacent hepatic tissue inflammation, n (%) | | | 0.21 | 1.58 |
| No | 46 (19.4%) | 72 (30.4%) | | |
| Yes | 56 (23.6%) | 63 (26.6%) | | |
| Pathologic T stage, n (%) | | | 0.29 | 1.10 |
| T1&T2 | 135 (36.4%) | 143 (38.5%) | | |
| T3&T4 | 51 (13.7%) | 42 (11.3%) | | |
| Pathologic N stage, n (%) | | | 1.00 | <0.001 |
| N0 | 125 (48.4%) | 129 (50%) | | |
| N1 | 2 (0.8%) | 2 (0.8%) | | |
| Pathologic M stage, n (%) | | | 0.62 | 0.24 |
| M0 | 134 (49.3%) | 134 (49.3%) | | |
| M1 | 3 (1.1%) | 1 (0.4%) | | |

## Slide 9
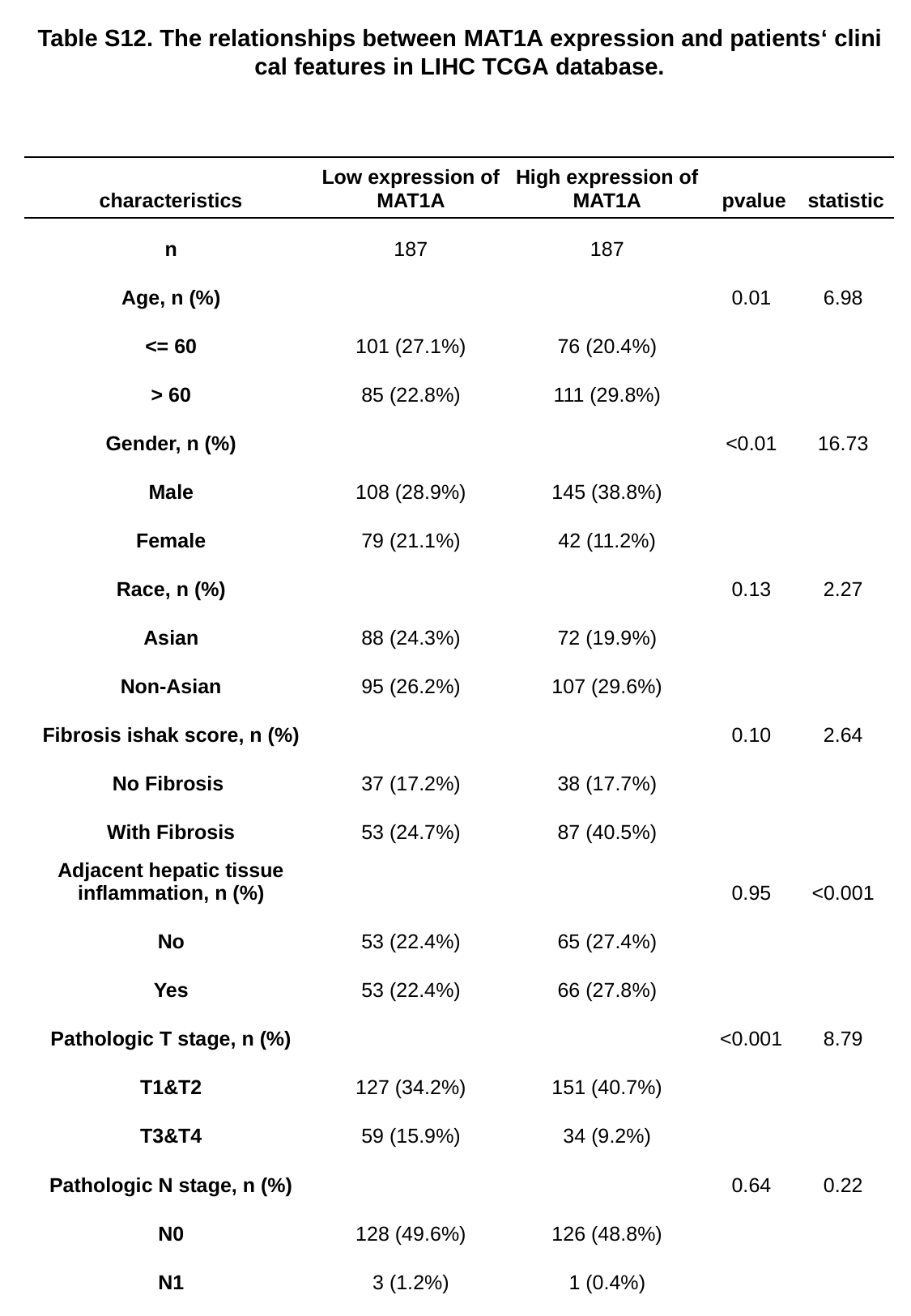

Table S12. The relationships between MAT1A expression and patients‘ clinical features in LIHC TCGA database.
| characteristics | Low expression of MAT1A | High expression of MAT1A | pvalue | statistic |
| --- | --- | --- | --- | --- |
| n | 187 | 187 | | |
| Age, n (%) | | | 0.01 | 6.98 |
| <= 60 | 101 (27.1%) | 76 (20.4%) | | |
| > 60 | 85 (22.8%) | 111 (29.8%) | | |
| Gender, n (%) | | | <0.01 | 16.73 |
| Male | 108 (28.9%) | 145 (38.8%) | | |
| Female | 79 (21.1%) | 42 (11.2%) | | |
| Race, n (%) | | | 0.13 | 2.27 |
| Asian | 88 (24.3%) | 72 (19.9%) | | |
| Non-Asian | 95 (26.2%) | 107 (29.6%) | | |
| Fibrosis ishak score, n (%) | | | 0.10 | 2.64 |
| No Fibrosis | 37 (17.2%) | 38 (17.7%) | | |
| With Fibrosis | 53 (24.7%) | 87 (40.5%) | | |
| Adjacent hepatic tissue inflammation, n (%) | | | 0.95 | <0.001 |
| No | 53 (22.4%) | 65 (27.4%) | | |
| Yes | 53 (22.4%) | 66 (27.8%) | | |
| Pathologic T stage, n (%) | | | <0.001 | 8.79 |
| T1&T2 | 127 (34.2%) | 151 (40.7%) | | |
| T3&T4 | 59 (15.9%) | 34 (9.2%) | | |
| Pathologic N stage, n (%) | | | 0.64 | 0.22 |
| N0 | 128 (49.6%) | 126 (48.8%) | | |
| N1 | 3 (1.2%) | 1 (0.4%) | | |
| Pathologic M stage, n (%) | | | 0.68 | 0.17 |
| M0 | 139 (51.1%) | 129 (47.4%) | | |
| M1 | 3 (1.1%) | 1 (0.4%) | | |

## Slide 10
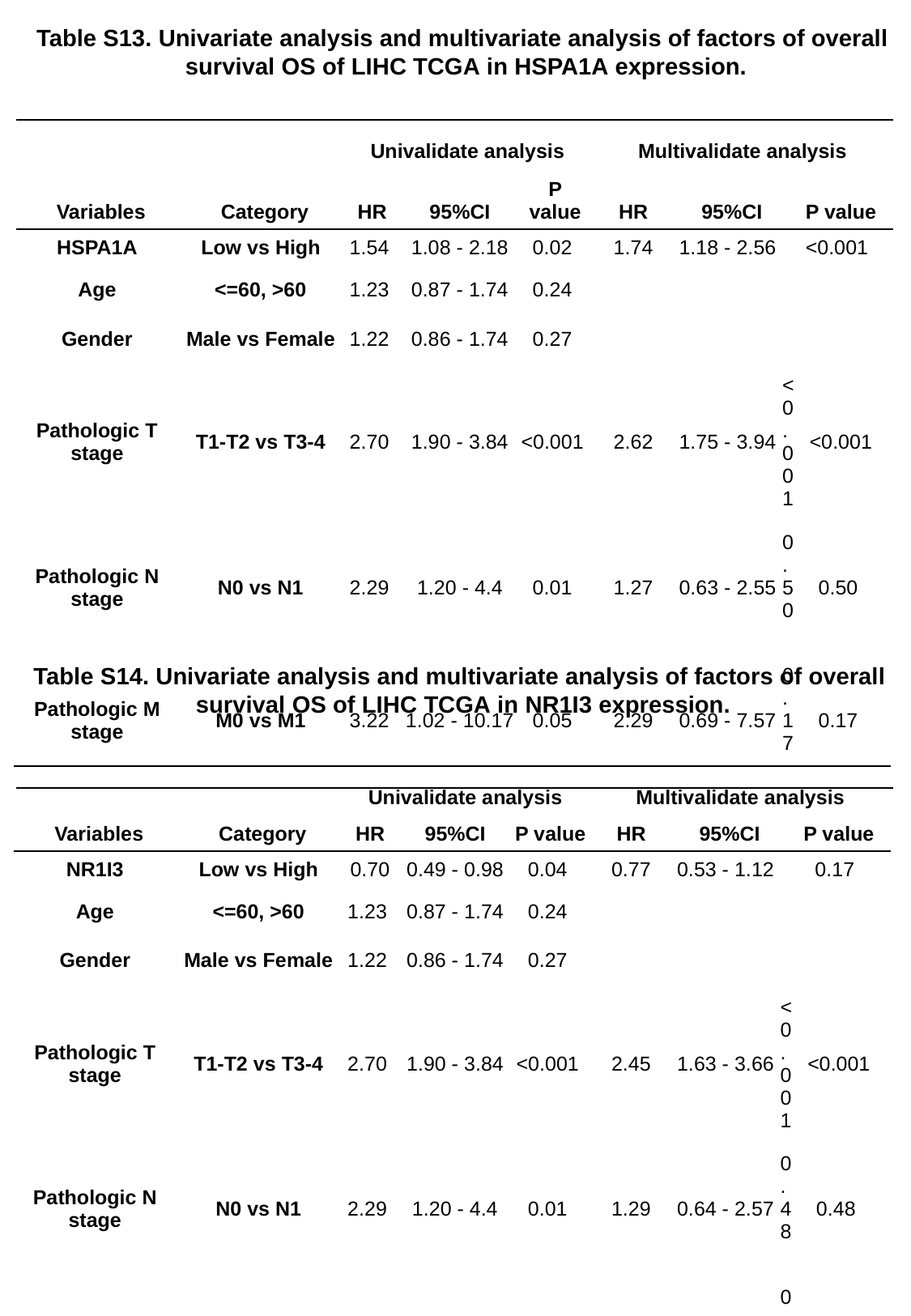

Table S13. Univariate analysis and multivariate analysis of factors of overall survival OS of LIHC TCGA in HSPA1A expression.
| | | | Univalidate analysis | | | Multivalidate analysis | | | |
| --- | --- | --- | --- | --- | --- | --- | --- | --- | --- |
| Variables | | Category | HR | 95%CI | P value | HR | 95%CI | | P value |
| HSPA1A | Low vs High | | 1.54 | 1.08 - 2.18 | 0.02 | 1.74 | 1.18 - 2.56 | <0.001 | |
| Age | <=60, >60 | | 1.23 | 0.87 - 1.74 | 0.24 | | | | |
| Gender | Male vs Female | | 1.22 | 0.86 - 1.74 | 0.27 | | | | |
| Pathologic T stage | T1-T2 vs T3-4 | | 2.70 | 1.90 - 3.84 | <0.001 | 2.62 | 1.75 - 3.94 | <0.001 | <0.001 |
| Pathologic N stage | N0 vs N1 | | 2.29 | 1.20 - 4.4 | 0.01 | 1.27 | 0.63 - 2.55 | 0.50 | 0.50 |
| Pathologic M stage | M0 vs M1 | | 3.22 | 1.02 - 10.17 | 0.05 | 2.29 | 0.69 - 7.57 | 0.17 | 0.17 |
Table S14. Univariate analysis and multivariate analysis of factors of overall survival OS of LIHC TCGA in NR1I3 expression.
| | | | Univalidate analysis | | | Multivalidate analysis | | | |
| --- | --- | --- | --- | --- | --- | --- | --- | --- | --- |
| Variables | | Category | HR | 95%CI | P value | HR | 95%CI | | P value |
| NR1I3 | Low vs High | | 0.70 | 0.49 - 0.98 | 0.04 | 0.77 | 0.53 - 1.12 | 0.17 | |
| Age | <=60, >60 | | 1.23 | 0.87 - 1.74 | 0.24 | | | | |
| Gender | Male vs Female | | 1.22 | 0.86 - 1.74 | 0.27 | | | | |
| Pathologic T stage | T1-T2 vs T3-4 | | 2.70 | 1.90 - 3.84 | <0.001 | 2.45 | 1.63 - 3.66 | <0.001 | <0.001 |
| Pathologic N stage | N0 vs N1 | | 2.29 | 1.20 - 4.4 | 0.01 | 1.29 | 0.64 - 2.57 | 0.48 | 0.48 |
| Pathologic M stage | M0 vs M1 | | 3.22 | 1.02 - 10.17 | 0.05 | 1.65 | 0.5 - 5.38 | 0.41 | 0.41 |

## Slide 11
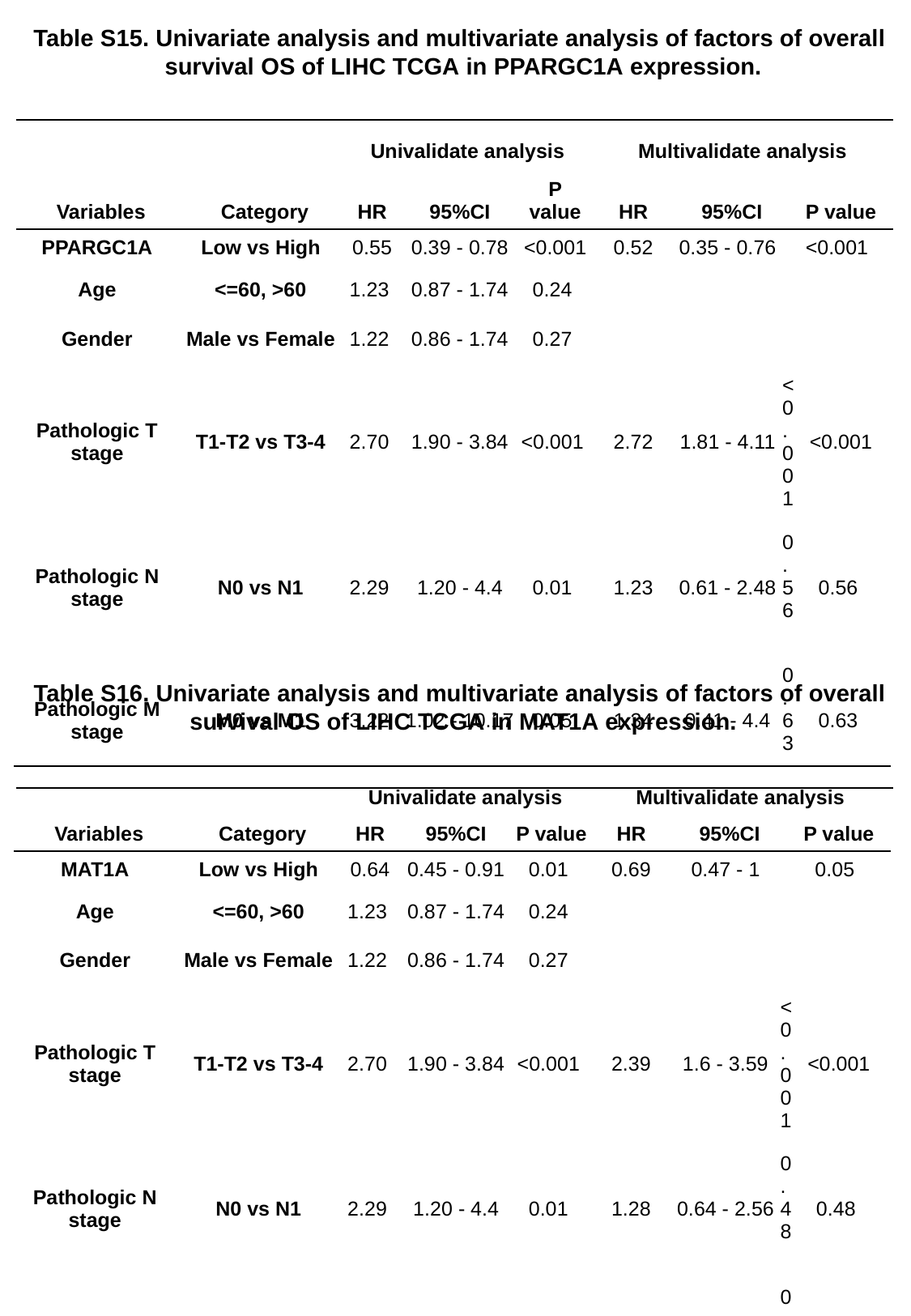

Table S15. Univariate analysis and multivariate analysis of factors of overall survival OS of LIHC TCGA in PPARGC1A expression.
| | | | Univalidate analysis | | | Multivalidate analysis | | | |
| --- | --- | --- | --- | --- | --- | --- | --- | --- | --- |
| Variables | | Category | HR | 95%CI | P value | HR | 95%CI | | P value |
| PPARGC1A | Low vs High | | 0.55 | 0.39 - 0.78 | <0.001 | 0.52 | 0.35 - 0.76 | <0.001 | |
| Age | <=60, >60 | | 1.23 | 0.87 - 1.74 | 0.24 | | | | |
| Gender | Male vs Female | | 1.22 | 0.86 - 1.74 | 0.27 | | | | |
| Pathologic T stage | T1-T2 vs T3-4 | | 2.70 | 1.90 - 3.84 | <0.001 | 2.72 | 1.81 - 4.11 | <0.001 | <0.001 |
| Pathologic N stage | N0 vs N1 | | 2.29 | 1.20 - 4.4 | 0.01 | 1.23 | 0.61 - 2.48 | 0.56 | 0.56 |
| Pathologic M stage | M0 vs M1 | | 3.22 | 1.02 - 10.17 | 0.05 | 1.34 | 0.41 - 4.4 | 0.63 | 0.63 |
Table S16. Univariate analysis and multivariate analysis of factors of overall survival OS of LIHC TCGA in MAT1A expression.
| | | | Univalidate analysis | | | Multivalidate analysis | | | |
| --- | --- | --- | --- | --- | --- | --- | --- | --- | --- |
| Variables | | Category | HR | 95%CI | P value | HR | 95%CI | | P value |
| MAT1A | Low vs High | | 0.64 | 0.45 - 0.91 | 0.01 | 0.69 | 0.47 - 1 | 0.05 | |
| Age | <=60, >60 | | 1.23 | 0.87 - 1.74 | 0.24 | | | | |
| Gender | Male vs Female | | 1.22 | 0.86 - 1.74 | 0.27 | | | | |
| Pathologic T stage | T1-T2 vs T3-4 | | 2.70 | 1.90 - 3.84 | <0.001 | 2.39 | 1.6 - 3.59 | <0.001 | <0.001 |
| Pathologic N stage | N0 vs N1 | | 2.29 | 1.20 - 4.4 | 0.01 | 1.28 | 0.64 - 2.56 | 0.48 | 0.48 |
| Pathologic M stage | M0 vs M1 | | 3.22 | 1.02 - 10.17 | 0.05 | 1.61 | 0.49 - 5.26 | 0.43 | 0.43 |

## Slide 12
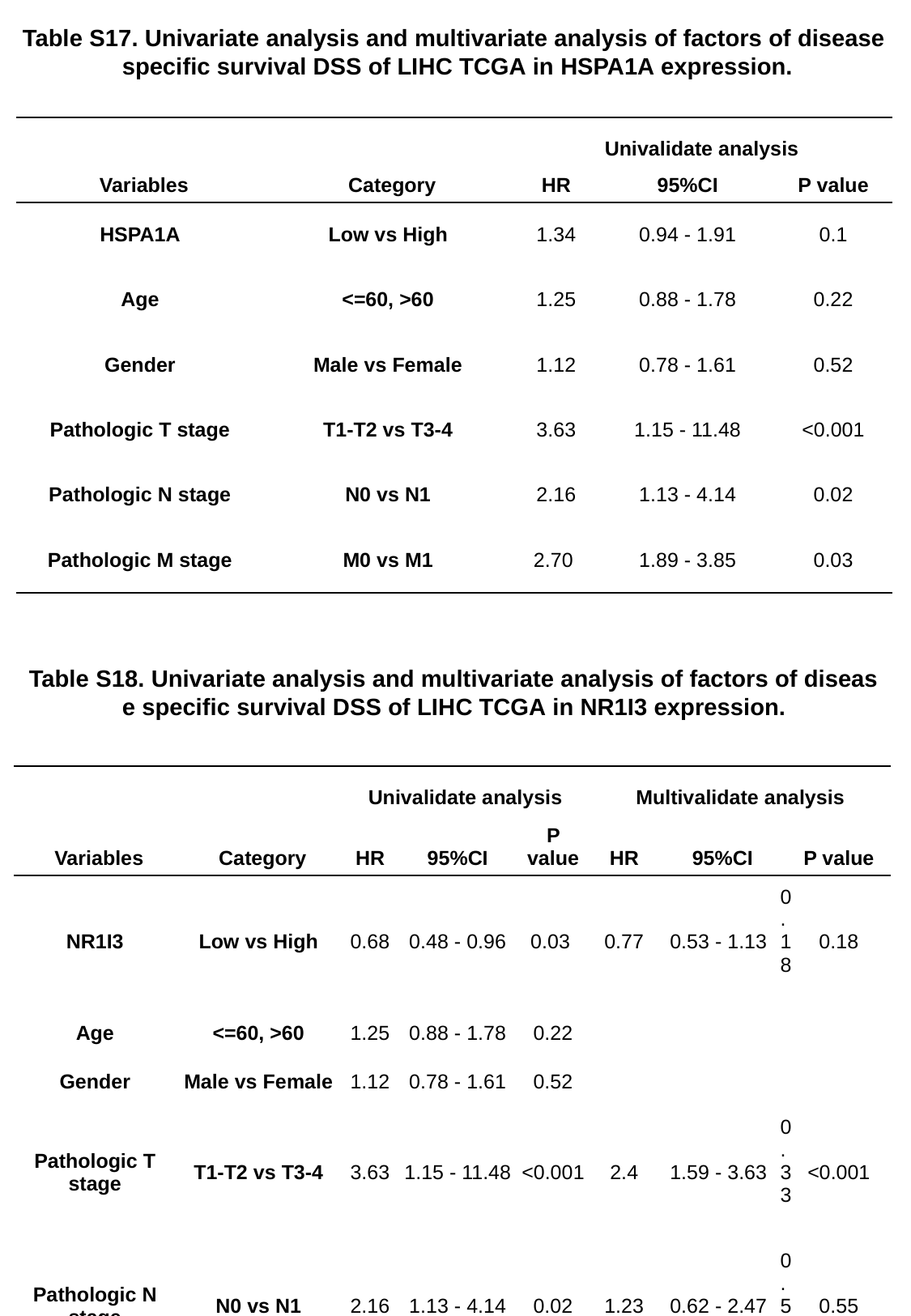

Table S17. Univariate analysis and multivariate analysis of factors of disease specific survival DSS of LIHC TCGA in HSPA1A expression.
| | | | Univalidate analysis | | |
| --- | --- | --- | --- | --- | --- |
| Variables | | Category | HR | 95%CI | P value |
| HSPA1A | Low vs High | | 1.34 | 0.94 - 1.91 | 0.1 |
| Age | <=60, >60 | | 1.25 | 0.88 - 1.78 | 0.22 |
| Gender | Male vs Female | | 1.12 | 0.78 - 1.61 | 0.52 |
| Pathologic T stage | T1-T2 vs T3-4 | | 3.63 | 1.15 - 11.48 | <0.001 |
| Pathologic N stage | N0 vs N1 | | 2.16 | 1.13 - 4.14 | 0.02 |
| Pathologic M stage | M0 vs M1 | | 2.70 | 1.89 - 3.85 | 0.03 |
Table S18. Univariate analysis and multivariate analysis of factors of disease specific survival DSS of LIHC TCGA in NR1I3 expression.
| | | | Univalidate analysis | | | Multivalidate analysis | | | |
| --- | --- | --- | --- | --- | --- | --- | --- | --- | --- |
| Variables | | Category | HR | 95%CI | P value | HR | 95%CI | | P value |
| NR1I3 | Low vs High | | 0.68 | 0.48 - 0.96 | 0.03 | 0.77 | 0.53 - 1.13 | 0.18 | 0.18 |
| Age | <=60, >60 | | 1.25 | 0.88 - 1.78 | 0.22 | | | | |
| Gender | Male vs Female | | 1.12 | 0.78 - 1.61 | 0.52 | | | | |
| Pathologic T stage | T1-T2 vs T3-4 | | 3.63 | 1.15 - 11.48 | <0.001 | 2.4 | 1.59 - 3.63 | 0.33 | <0.001 |
| Pathologic N stage | N0 vs N1 | | 2.16 | 1.13 - 4.14 | 0.02 | 1.23 | 0.62 - 2.47 | 0.55 | 0.55 |
| Pathologic M stage | M0 vs M1 | | 2.70 | 1.89 - 3.85 | 0.03 | 1.80 | 0.55 - 5.91 | <0.002 | 0.33 |

## Slide 13
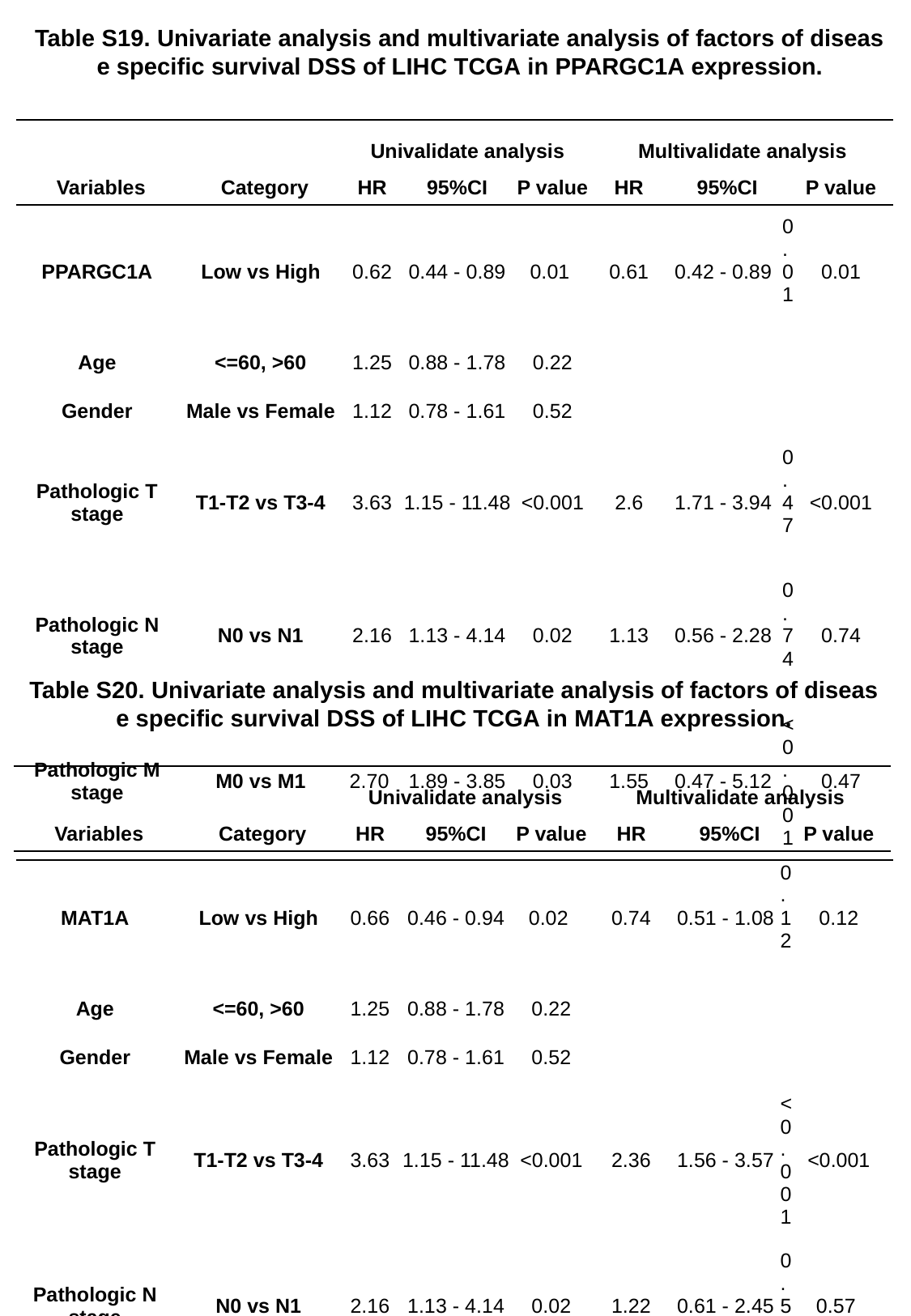

Table S19. Univariate analysis and multivariate analysis of factors of disease specific survival DSS of LIHC TCGA in PPARGC1A expression.
| | | | Univalidate analysis | | | Multivalidate analysis | | | |
| --- | --- | --- | --- | --- | --- | --- | --- | --- | --- |
| Variables | | Category | HR | 95%CI | P value | HR | 95%CI | | P value |
| PPARGC1A | Low vs High | | 0.62 | 0.44 - 0.89 | 0.01 | 0.61 | 0.42 - 0.89 | 0.01 | 0.01 |
| Age | <=60, >60 | | 1.25 | 0.88 - 1.78 | 0.22 | | | | |
| Gender | Male vs Female | | 1.12 | 0.78 - 1.61 | 0.52 | | | | |
| Pathologic T stage | T1-T2 vs T3-4 | | 3.63 | 1.15 - 11.48 | <0.001 | 2.6 | 1.71 - 3.94 | 0.47 | <0.001 |
| Pathologic N stage | N0 vs N1 | | 2.16 | 1.13 - 4.14 | 0.02 | 1.13 | 0.56 - 2.28 | 0.74 | 0.74 |
| Pathologic M stage | M0 vs M1 | | 2.70 | 1.89 - 3.85 | 0.03 | 1.55 | 0.47 - 5.12 | <0.001 | 0.47 |
Table S20. Univariate analysis and multivariate analysis of factors of disease specific survival DSS of LIHC TCGA in MAT1A expression.
| | | | Univalidate analysis | | | Multivalidate analysis | | | |
| --- | --- | --- | --- | --- | --- | --- | --- | --- | --- |
| Variables | | Category | HR | 95%CI | P value | HR | 95%CI | | P value |
| MAT1A | Low vs High | | 0.66 | 0.46 - 0.94 | 0.02 | 0.74 | 0.51 - 1.08 | 0.12 | 0.12 |
| Age | <=60, >60 | | 1.25 | 0.88 - 1.78 | 0.22 | | | | |
| Gender | Male vs Female | | 1.12 | 0.78 - 1.61 | 0.52 | | | | |
| Pathologic T stage | T1-T2 vs T3-4 | | 3.63 | 1.15 - 11.48 | <0.001 | 2.36 | 1.56 - 3.57 | <0.001 | <0.001 |
| Pathologic N stage | N0 vs N1 | | 2.16 | 1.13 - 4.14 | 0.02 | 1.22 | 0.61 - 2.45 | 0.57 | 0.57 |
| Pathologic M stage | M0 vs M1 | | 2.70 | 1.89 - 3.85 | 0.03 | 1.80 | 0.55 - 5.89 | 0.33 | 0.33 |

## Slide 14
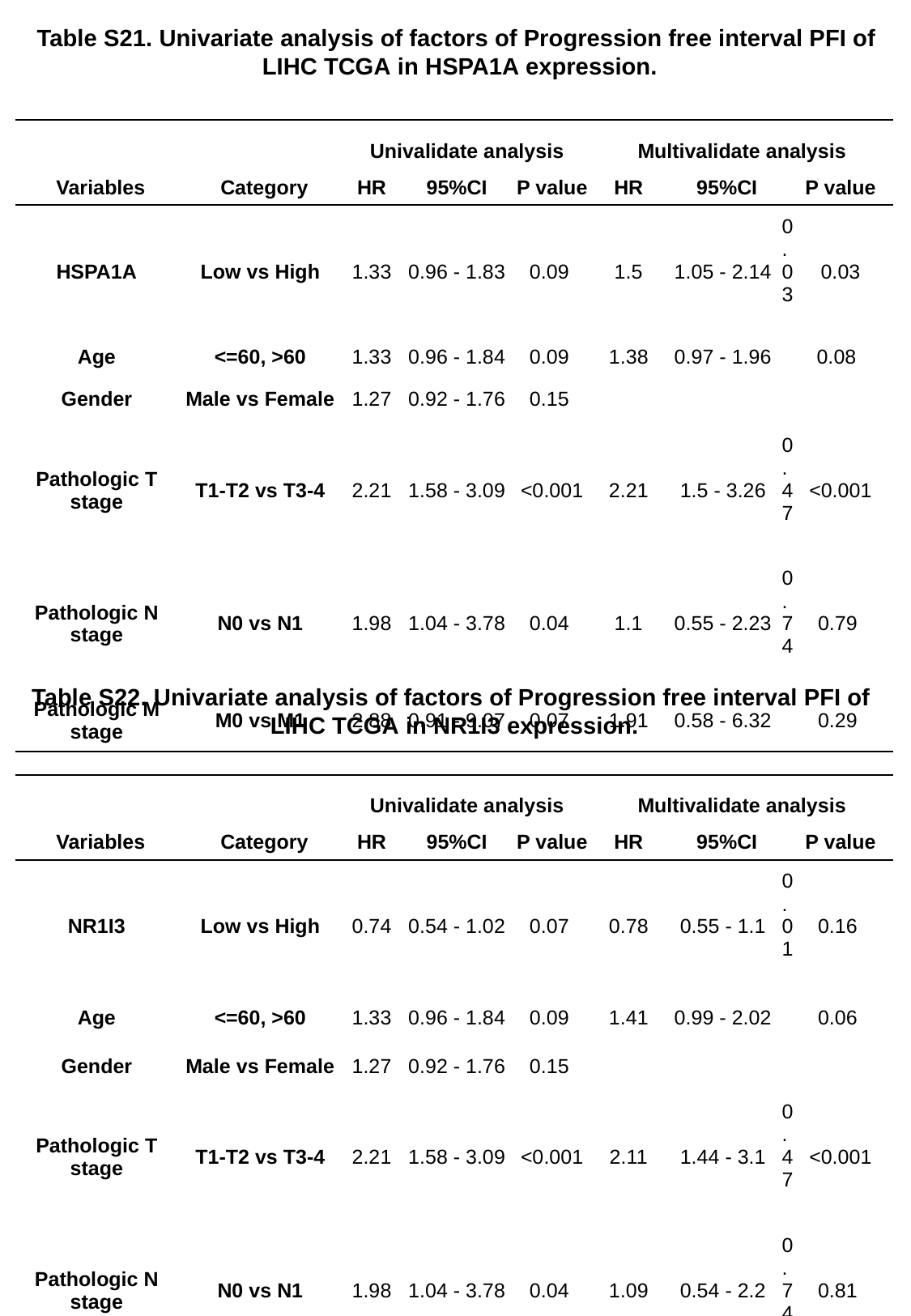

Table S21. Univariate analysis of factors of Progression free interval PFI of LIHC TCGA in HSPA1A expression.
| | | | Univalidate analysis | | | Multivalidate analysis | | | |
| --- | --- | --- | --- | --- | --- | --- | --- | --- | --- |
| Variables | | Category | HR | 95%CI | P value | HR | 95%CI | | P value |
| HSPA1A | Low vs High | | 1.33 | 0.96 - 1.83 | 0.09 | 1.5 | 1.05 - 2.14 | 0.03 | 0.03 |
| Age | <=60, >60 | | 1.33 | 0.96 - 1.84 | 0.09 | 1.38 | 0.97 - 1.96 | 0.08 | |
| Gender | Male vs Female | | 1.27 | 0.92 - 1.76 | 0.15 | | | | |
| Pathologic T stage | T1-T2 vs T3-4 | | 2.21 | 1.58 - 3.09 | <0.001 | 2.21 | 1.5 - 3.26 | 0.47 | <0.001 |
| Pathologic N stage | N0 vs N1 | | 1.98 | 1.04 - 3.78 | 0.04 | 1.1 | 0.55 - 2.23 | 0.74 | 0.79 |
| Pathologic M stage | M0 vs M1 | | 2.88 | 0.91 - 9.07 | 0.07 | 1.91 | 0.58 - 6.32 | | 0.29 |
Table S22. Univariate analysis of factors of Progression free interval PFI of LIHC TCGA in NR1I3 expression.
| | | | Univalidate analysis | | | Multivalidate analysis | | | |
| --- | --- | --- | --- | --- | --- | --- | --- | --- | --- |
| Variables | | Category | HR | 95%CI | P value | HR | 95%CI | | P value |
| NR1I3 | Low vs High | | 0.74 | 0.54 - 1.02 | 0.07 | 0.78 | 0.55 - 1.1 | 0.01 | 0.16 |
| Age | <=60, >60 | | 1.33 | 0.96 - 1.84 | 0.09 | 1.41 | 0.99 - 2.02 | | 0.06 |
| Gender | Male vs Female | | 1.27 | 0.92 - 1.76 | 0.15 | | | | |
| Pathologic T stage | T1-T2 vs T3-4 | | 2.21 | 1.58 - 3.09 | <0.001 | 2.11 | 1.44 - 3.1 | 0.47 | <0.001 |
| Pathologic N stage | N0 vs N1 | | 1.98 | 1.04 - 3.78 | 0.04 | 1.09 | 0.54 - 2.2 | 0.74 | 0.81 |
| Pathologic M stage | M0 vs M1 | | 2.88 | 0.91 - 9.07 | 0.07 | 1.44 | 0.44 - 4.76 | | 0.55 |

## Slide 15
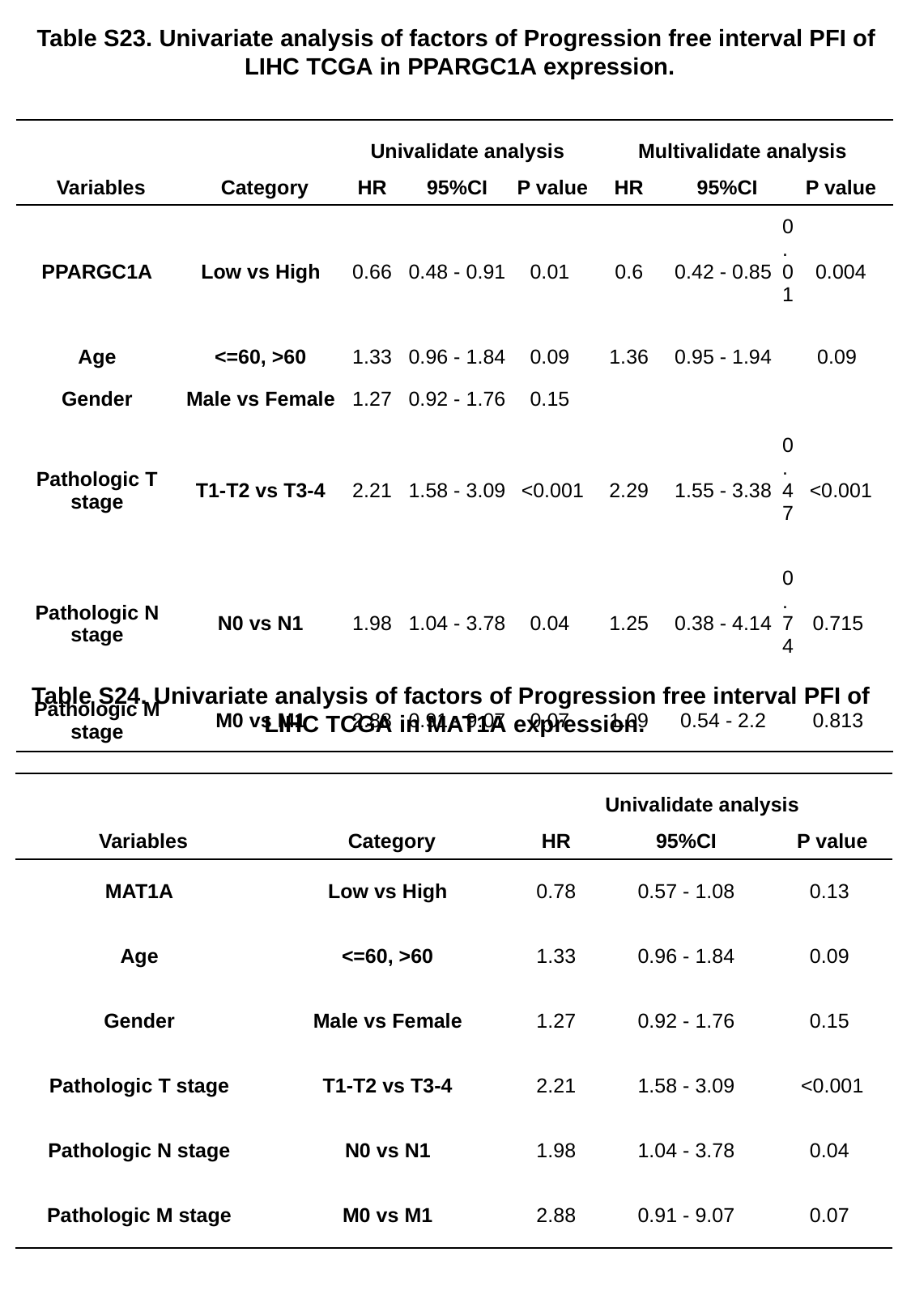

Table S23. Univariate analysis of factors of Progression free interval PFI of LIHC TCGA in PPARGC1A expression.
| | | | Univalidate analysis | | | Multivalidate analysis | | | |
| --- | --- | --- | --- | --- | --- | --- | --- | --- | --- |
| Variables | | Category | HR | 95%CI | P value | HR | 95%CI | | P value |
| PPARGC1A | Low vs High | | 0.66 | 0.48 - 0.91 | 0.01 | 0.6 | 0.42 - 0.85 | 0.01 | 0.004 |
| Age | <=60, >60 | | 1.33 | 0.96 - 1.84 | 0.09 | 1.36 | 0.95 - 1.94 | 0.09 | |
| Gender | Male vs Female | | 1.27 | 0.92 - 1.76 | 0.15 | | | | |
| Pathologic T stage | T1-T2 vs T3-4 | | 2.21 | 1.58 - 3.09 | <0.001 | 2.29 | 1.55 - 3.38 | 0.47 | <0.001 |
| Pathologic N stage | N0 vs N1 | | 1.98 | 1.04 - 3.78 | 0.04 | 1.25 | 0.38 - 4.14 | 0.74 | 0.715 |
| Pathologic M stage | M0 vs M1 | | 2.88 | 0.91 - 9.07 | 0.07 | 1.09 | 0.54 - 2.2 | | 0.813 |
Table S24. Univariate analysis of factors of Progression free interval PFI of LIHC TCGA in MAT1A expression.
| | | | Univalidate analysis | | |
| --- | --- | --- | --- | --- | --- |
| Variables | | Category | HR | 95%CI | P value |
| MAT1A | Low vs High | | 0.78 | 0.57 - 1.08 | 0.13 |
| Age | <=60, >60 | | 1.33 | 0.96 - 1.84 | 0.09 |
| Gender | Male vs Female | | 1.27 | 0.92 - 1.76 | 0.15 |
| Pathologic T stage | T1-T2 vs T3-4 | | 2.21 | 1.58 - 3.09 | <0.001 |
| Pathologic N stage | N0 vs N1 | | 1.98 | 1.04 - 3.78 | 0.04 |
| Pathologic M stage | M0 vs M1 | | 2.88 | 0.91 - 9.07 | 0.07 |
